# Nutrient Imbalance of the Host Plant for Larvae of the Pale Grass Blue Butterfly May Mediate the Field Effect of Low-Dose Radiation Exposure in Fukushima: Dose-Dependent Changes in the Sodium Content

**DOI:** 10.3390/insects12020149

**Published:** 2021-02-09

**Authors:** Ko Sakauchi, Wataru Taira, Mariko Toki, Masakazu Tsuhako, Kazuo Umetsu, Joji M. Otaki

**Affiliations:** 1 The BCPH Unit of Molecular Physiology, Department of Chemistry, Biology and Marine Science, Faculty of Science, University of the Ryukyus, Okinawa 903-0213, Japan; 2 Center for Research Advancement and Collaboration, University of the Ryukyus, Okinawa 903-0213, Japan; 3 Department of Forensic Medicine, Yamagata University, School of Medicine, Yamagata 990-9585, Japan; kumetsu@med.id.yamagata-u.ac.jp

**Keywords:** Fukushima nuclear accident, lycaenid butterfly, creeping wood sorrel, nutrient content, *Oxalis corniculata*, radiation stress

## Abstract

**Simple Summary:**

The pale grass blue butterfly has been used to monitor the biological impacts of the Fukushima nuclear accident because this butterfly is sensitive to low-dose radioactive pollution in the field. However, the butterfly has been shown to be highly tolerant to radioactive cesium (^137^Cs), the major radionuclide in Fukushima, in an artificial diet in laboratory experiments. This field-laboratory paradox may be explained by the field-effect hypothesis; the host plant may change its nutrient contents in response to radiation stress. Leaves from Tohoku (mostly polluted areas including Fukushima) showed significantly lower sodium contents than those from Niigata. In Tohoku samples, an increase in the radioactivity concentration of cesium (^137^Cs) in leaves or in the ground radiation dose was accompanied by a decrease in the sodium content. The sodium content appeared to be related to other nutrient factors. Thus, the sodium imbalance of the plant may be caused by radiation stress, and this imbalance may be one of the reasons that this monophagous butterfly showed high mortality and morphological abnormalities in the field shortly after the accident in Fukushima.

**Abstract:**

The pale grass blue butterfly *Zizeeria maha* is sensitive to low-dose radioactive pollution from the Fukushima nuclear accident in the field but is also highly tolerant to radioactive cesium (^137^Cs) in an artificial diet in laboratory experiments. To resolve this field-laboratory paradox, we hypothesize that the butterfly shows vulnerability in the field through biochemical changes in the larval host plant, the creeping wood sorrel *Oxalis corniculata*, in response to radiation stress. To test this field-effect hypothesis, we examined nutrient contents in the host plant leaves from Tohoku (mostly polluted areas including Fukushima), Niigata, and Kyushu, Japan. Leaves from Tohoku showed significantly lower sodium and lipid contents than those from Niigata. In the Tohoku samples, the sodium content (but not the lipid content) was significantly negatively correlated with the radioactivity concentration of cesium (^137^Cs) in leaves and with the ground radiation dose. The sodium content was also correlated with other nutrient factors. These results suggest that the sodium imbalance of the plant may be caused by radiation stress and that this nutrient imbalance may be one of the reasons that this monophagous butterfly showed high mortality and morphological abnormalities in the field shortly after the accident in Fukushima.

## 1. Introduction

Upon the Fukushima nuclear accident in March 2011, a massive amount of radioactive materials was released from the Fukushima Dai-ichi Nuclear Power Plant to the surrounding environment in the Tohoku district, Japan [1,2,3,4,5,6,7,8,9,10]. Possible effects of this radioactive pollution on human health have been the central matter of concern of scientists, physicians, and the public; however, not surprisingly, studies that show causality of the Fukushima nuclear accident to human health problems are relatively scarce [11,12,13,14,15,16]. In contrast, studies on the possible effects of radioactive pollution in Fukushima on wild animals and plants have gradually accumulated in the literature. Among them, possible effects on hematological and developmental factors in wild Japanese monkeys [17,18,19] may be of high importance because Japanese monkeys are primates living relatively close to humans in Fukushima. Other studies on mammals such as field mice and cattle showed either the possible effects or null effects of radioactive pollution in Fukushima [20,21,22,23,24,25,26,27,28,29,30,31]. Among other vertebrates, some birds, such as barn swallows and goshawks, appeared to be affected by radioactive pollution in Fukushima [32,33,34].

Similarly, many invertebrate species are reported to be affected. The number of invertebrate species has decreased in intertidal areas [35] and on land [36,37]. Among invertebrates, insects have been used to monitor possible effects of radioactive contamination in environments [36,37,38,39,40,41,42,43,44,45]. The severe morphological abnormalities of gall-forming aphids found in Fukushima but not in uncontaminated areas are striking [39], although the low-dose exposure experiments did not produce any morphological abnormalities or fatal results [40]. Furthermore, possible responses of several species of plants to radioactive contamination have been reported [46,47,48,49,50,51,52,53,54,55,56,57]. As herbivorous insects totally depend on the quality and quantity of available edible plants, ecological interactions between insects and plants may be disrupted if insects’ host plants are affected genetically and physiologically by radioactive pollution.

Among insects, the pale grass blue butterfly *Zizeeria maha* (Lepidoptera, Lycaenidae) is an important organism that has been studied since immediately after the Fukushima nuclear accident [38,58,59,60,61,62,63,64,65,66,67,68,69,70,71,72,73,74,75,76]. This butterfly has many advantages both as a sampling animal for field work and as a laboratory animal [77]. Thus, many “field-based” data have been accumulated for this butterfly. Our field-based data were obtained from field butterfly sampling, morphological and inheritance examinations, and other experiments, together demonstrating that the Fukushima nuclear accident caused high mortality and morphological abnormality rates in this butterfly in the field. One of the important field-based experiments was the so-called internal exposure experiment, in which larvae in Okinawa without previous exposure to radionuclides from the Fukushima nuclear accident were fed contaminated leaves from Fukushima, resulting in high mortality and abnormality rates [38,60]. This experiment was repeated in additional studies using the same species [61,62,63], and a similar but more rigorous experiment was conducted using the cabbage white butterfly [45], confirming the results of the previous internal exposure experiments. Although the exposure level of the pale grass blue butterfly has not been rigorously determined, the butterfly’s vulnerability to radioactive contaminants does not fit the conventional dosimetric view that insects are highly tolerant to radioactive exposure. Remarkably, our “laboratory-based” data have revealed that the pale grass blue butterfly was highly tolerant to radioactive cesium (^137^Cs), the major radioactive contaminant in leaves from Fukushima, when fed an artificial diet [72]. This result is reminiscent of the case of gall-forming aphids [39,40].

To resolve this field-laboratory paradox, field effects have been proposed [71]. Field effects are the effects that occur in the field but not necessarily in controlled laboratory experiments. In the case of this butterfly, the field effects may work through the host plant leaves that are exposed to radioactive contaminants despite the plant not showing an abnormal phenotype. For example, radiation stress may cause plants to synthesize insect repellent or toxicant chemicals. Alternatively, the plant may lower the production of some vitamins or nutrients that are essential for larvae to grow and metamorphosize. The larvae of this butterfly exclusively eat the creeping wood sorrel *Oxalis corniculata* (Oxalidales: Oxalidaceae), completely depending on the host plant leaves for their nutrients [78,79,80]. Virtually no alternative food is available for larvae. Accordingly, we hypothesized that if the nutritional values of the host plant leaves are affected by radioactive materials, the larvae may starve, resulting in malformations or death.

As a first step to examine the feasibility of this field-effect hypothesis, we analyzed the nutrient contents of the host plant leaves. We collected leaves of the creeping wood sorrel from the Tohoku district, including Fukushima. For comparison, we collected leaves from Niigata and Kyushu. Tohoku and Niigata are located at similar latitudes, but Niigata is much less polluted with radioactive materials from the Fukushima Dai-ichi Nuclear Power Plant than Tohoku. Thus, Niigata served as an excellent “control”. The pollution level of Kyushu is also minimal, and Kyushu served as an additional control, although it is located at a different latitude.

## 2. Materials and Methods

### 2.1. Plant and Butterfly

**Figure 1:**
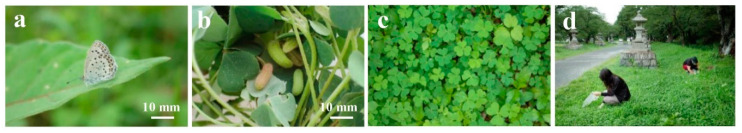
The pale grass blue butterfly and the creeping wood sorrel. (**a**) An adult butterfly in Namie Town, Fukushima Prefecture. (**b**) Larvae on the leaves of the plant in the laboratory. (**c**) Cluster of the plant in Nishihara Town, Okinawa Prefecture. (**d**) Collecting leaves for this study alongside a walking road in Soma City, Fukushima Prefecture.

The life history of the pale grass blue butterfly *Zizeeria maha* (Figure 1a,b) is totally dependent on its host plant, the creeping wood sorrel *Oxalis corniculata* (Oxalidales: Oxalidaceae) (Figure 1c) [77,78,79,80]. The origin of this plant appears to be Southeast Asia [81]. This plant is distributed throughout Japan and is found in human-living environments, including residential areas, city parks, riversides, and agricultural villages (Figure 1d). As this butterfly has a limited dispersion range [82] and is monophagous [77,78,79,80], the plant and the butterfly are often found simultaneously at the same sites in human-living environments [70]. This plant contains oxalic acid, which may function to eradicate insects other than the pale grass blue butterfly [83].

### 2.2. Sampling and Temperature Data

**Figure 2:**
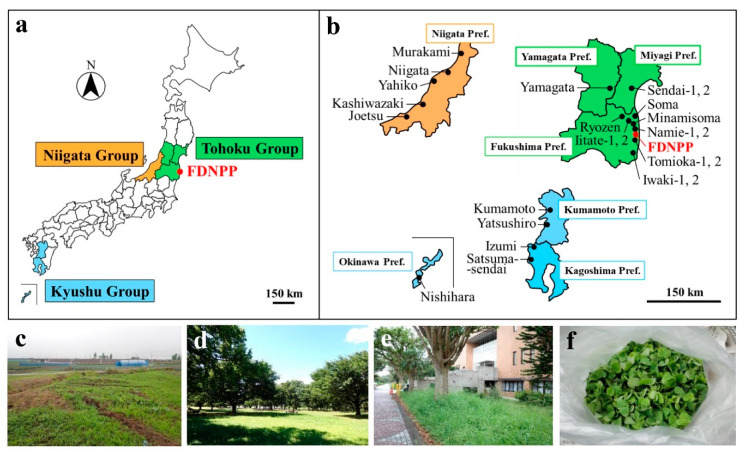
Field sampling localities and examples of landscapes of collection sites. (**a**) A Japan-wide map with prefectural borders. Prefectures where the plant samples were collected are colored. FDNPP: Fukushima Dai-ichi Nuclear Power Plant. (**b**) Collection localities. (**c**) Sendai-2 from the Tohoku group. (**d**) Murakami from the Niigata group. (**e**) Nishihara from the Kyushu group. (**f**) Sampled and stem-separated leaves of *Oxalis corniculata*.

We sampled and examined the leaves of this plant species from contaminated and uncontaminated fields in Japan. No sampling sites were affected by tsunamis when the Great East Japan Earthquake occurred. The leaves of this plant were collected from field sites in November 2016 (Tohoku), December 2016 (Kyushu), July 2018 (Niigata), and July 2020 (Tohoku) (Figure 2a,b; Table S1). The leaves were sampled from 24 localities in total, and samples were categorized into three locality groups: the Tohoku group, the Niigata group, and the Kyushu group (Figure 2a,b; Table S1). Analytical results of the nutrient contents and ^137^Cs and ^40^K radioactivity concentrations are compiled in Tables S2–S4. Nishihara samples were obtained from the same site twice in the morning (Nishihara-AM) and in the afternoon (Nishihara-PM) on the same day to check if there was any change within a day. There were indeed changes within a day, but the data showed that the changes were not very large in comparison with the data from other collection sites (Table S4). We thus combined the morning and afternoon data as the data from a single collection site for statistical analyses.

Temperature data were obtained from the nearest meteorological observatories of the Japan Meteorological Agency (http://www.jma.go.jp/jma/indexe.html) (accessed on 8 October 2020). To do so, temperature data for every hour were referred to, and the temperature at the starting time of leaf collection was obtained, considering the relatively quick (and possibly adaptive) changes of the Nishihara data within a day. When an observatory was not found in the collection locality, temperature data were obtained from the nearest observatory. In this study, soil was not sampled. Soil composition may affect biochemical contents in plants under radiation stress, but this possibility was not examined in this study.

### 2.3. Sampling Procedure

Leaf sampling was mostly conducted by three persons at the sampling sites until the leaf mass reached or exceeded approximately 130 g per site (Figure 1d). At the sampling sites, the host plant was hand-picked carefully from the stem, and leaves were further isolated from the stem manually. Dead or dying leaves and other contaminants, such as leaves of other plant species, were eliminated manually. Leaves with insect bites or with yellowish parts were eliminated. Physically broken leaves were also eliminated. The high-quality fresh leaf samples (ca. 130 g) were obtained this way (Figure 2f). These leaf samples were split into two groups (100 g for nutrient analyses and 30 g for radionuclide analyses). The leaf samples for nutrient analyses were washed twice with bottled natural mineral water, Evian (Evian les Bains, France). After the water was drained, the leaves were pat dried and stored in a plastic bag, and the bag was sealed. These leaf bags were packed in a cardboard box and sent to the Japan Functional Food Analysis and Research Center (Fukuoka, Japan) under refrigeration (unfrozen) conditions (0–10 °C) within the sampling day. The package arrived at the Center in Fukuoka within 2 days, upon which the leaf samples were visually checked again and confirmed that they were reasonably green and viable. When a small number of yellowish leaves were found at that point, they were hand-picked to be eliminated. The leaf samples for radionuclide analysis (to obtain the radioactivity concentrations of ^137^Cs and ^40^K) were stored in a box with desiccant and brought to the laboratory at the University of the Ryukyus, Okinawa, Japan. For both sample groups, leaves were kept in the dark under refrigeration (unfrozen) conditions (0–10 °C) as much as possible while at work.

### 2.4. Measurements of Ground Radiation Dose

Ground radiation doses (more precisely, ground radiation dose rates [μSv/h]) were measured using a Hitachi Aloka Medical TCS-172B scintillation survey meter (Tokyo, Japan) for 90 s at 3–7 points in the area of leaf collection with the probe set at 0 cm from the ground surface. These measurements were then averaged. This survey meter was calibrated using a ^266^Ra radiation source (Japan Isotope Association). For the Nishihara samples, ground dose measurements were made for the PM samples but not for the AM samples. For the latter, the data for the former were used.

### 2.5. Nutrient Analyses

The leaves were subjected to standard analyses for nutrients in the Japan Functional Food Analysis and Research Center (Fukuoka, Japan). We used 100 g of leaves per site. Mass of a single trifoliate (note: a single leaf contains three leaflets) collected in Nishihara, Okinawa, in January 2021 was determined as 60.315 ± 24.306 mg (mean ± SD; *n* = 10) using a Mettler-Toledo MX5 Microbalance (Columbus, OH, USA). This means that 1658 leaves (ca. 1700 leaves) per site were subjected to the analyses.

The following seven nutritional factors were quantified: energy [kcal/100 g], protein content [g/100 g], lipid content [g/100 g], carbohydrate content [g/100 g], sodium content [mg/100 g], water content [g/100 g], and ash content [g/100 g]. All contents were indicated as per 100 g of wet mass of leaves. Measurements were made three times per sample for the Tohoku and Kyushu groups and twice per sample for the Niigata group using the same prepared samples. These nutritional values were determined to meet the Food Labeling Standards of the Food Labeling Act (Act No. 70 of 2013) of Japan. Protocols for analytical methods, including calculations of energy and carbohydrate content, were in accordance with Japan Food Standard Nutrient Contents Tables 2015 [84]. These data are compiled in Tables S2–S4, and the correlation analysis results are shown in Tables S5–S8.

Energy was calculated using the following energy conversion factors: 4 for protein, 9 for lipid, and 4 for carbohydrate. The protein content was determined by the Dumas method (nitrogen combustion method). Gaseous nitrogen released from the combustion of a leaf sample was measured, and the nitrogen content was converted to the protein content using a nitrogen-protein conversion factor of 6.25. For the lipid content analysis, an acid hydrolysis method was employed: samples were treated with hydrochloric acid to release lipid moieties from carbohydrates and proteins. Samples were then extracted by diethyl ether and petroleum ether. After solvent evaporation, the remaining mass was determined. The sodium content was determined by atomic absorption spectroscopy. The ash content was determined by the direct ashing method, in which organic materials were released as carbon dioxide by heating and the remaining samples were weighed. Ash was defined as mineral remnants of combustion containing sodium, potassium, calcium, magnesium, iron, phosphorus, and other minerals. The water content was determined by the hot-air vacuum drying method. The carbohydrate content was determined by the equation: 100 − (water + protein + lipid + ash). Due to this definition, these five macronutrient-related factors are compositional data.

### 2.6. Measurements of Radioactivity Concentrations

The radioactivity concentrations of ^137^Cs and ^40^K in leaves were analyzed separately from nutrient analyses. As ^137^Cs is the major anthropogenic radionuclide in Fukushima, the level of ^137^Cs was considered a good indicator of radioactive pollution. In contrast, ^40^K is a naturally occurring radionuclide. As ^40^K occurs in a defined ratio (0.0117%) with other potassium species, the level of ^40^K was considered a good indicator of the level of the entire potassium species. These data are compiled in Tables S2–S4. The ratio of Na to ^40^K (indicated as the Na/^40^K ratio hereafter) was calculated without unit conversions to avoid introducing assumptions for calculations.

In the laboratory, leaf samples were dried completely with desiccant, crushed into very small pieces, put in a cylindrical columnar plastic vial (15 mm in diameter and 50 mm in height) to make a 35-mm sample height, and subjected to measurements using two machines of Canberra GCW-4023 germanium semiconductor radiation detectors (Meriden, CT, USA) in the Center for Research Advancement and Collaboration, University of the Ryukyus, Okinawa, Japan. Measurements were continued to obtain both ^137^Cs and ^40^K signals until the error rate became less than 5% of the measured values. However, measurements were terminated after 7 days before reaching this level when radioactivity was low. The detection efficiency values of ^137^Cs (662 keV) were 14.56% and 14.61%, and those for ^40^K (1461 keV) were 5.727% and 6.890% for each of the two machines. The branching ratios were 85.1% for ^137^Cs and 10.7% for ^40^K [85]. The radioactivity concentrations of ^137^Cs and ^40^K at the collection dates were calculated from those of the measuring dates based on the fact that the decay began on 15 March 2011, and that the half-lives of ^137^Cs and ^40^K are 30.167 years and 1.251 × 10^9^ years, respectively [85]. The radioactivity concentration was expressed as the becquerel per kilogram [Bq/kg] of dried samples. These two machines were calibrated according to an NBL protocol (New Brunswick Laboratory, U.S. Department of Energy, Argonne, IL, USA). The results are compiled in Tables S2–S4, and their correlation analysis results are shown in Tables S5–S8. In the case of the leaf samples from Nishihara Town, Okinawa Prefecture, only the morning sample was subjected to radiation measurements, and it was assumed that the afternoon samples had the same radiation values. Radioactivity concentrations of ^137^Cs in Sendai-1 in the Tohoku group and all localities in the Niigata and Kyushu groups were below the detection limit under our experimental conditions. For statistical analyses, their ^137^Cs values were considered zero.

### 2.7. Statistical Analyses

The statistical software R, version 3.5.3 (The R foundation for Statistical Computing, Vienna, Austria, 2019) [86], was used to perform statistical analyses. For graphic representations, Microsoft Excel (Microsoft 365) was used. Two radiation-related variables (^137^Cs level in *Oxalis* leaves [Bq/kg] and ground dose [μSv/h]) and temperature were considered the input variables. Nutrient factors were considered the output variables.

For comparisons among the Tohoku, Niigata, and Kyushu groups, ANOVA (summary(aov(X - Y))) was performed, and when significant differences were obtained, the Tukey–Kramer test (package: multcomp, summary(glht(aov(X - Y), linfct=mcp(Y=“Tukey”)))) was performed to obtain the *p*-values for the two pairs. Two subgroups (high and low subgroups) were compared using Welch’s *t*-test (t.test(X, Y)) according to the results of the *F*-test (var.test(X, Y)). The coefficient of variation (CV) was calculated as the standard deviation (SD) divided by the mean, using Microsoft Excel (Table S9). The CV is a unitless indicator of relative variability. Distribution gaps were identified manually; the largest gap between the nearest two samples was considered as the gap dividing the high and low subgroups. However, in the case of the ^137^Cs radioactivity concentrations, the largest gap simply set aside a single largest sample. Thus, the second largest gap was used as the gap dividing the high and low subgroups. The Pearson correlation coefficients (*r* values) (cor.test(X, Y, method=“pearson”)) were obtained together with their associated *p*-values, and they were compiled as correlation matrices (Tables S5–S8). Normality was checked with the Shapiro–Wilk test (shapiro.test(X)); *p* > 0.001 was considered normally distributed. As a result, all factors were assumed to be normally distributed.

Since the five nutrient contents (i.e., protein, lipid, carbohydrate, water, and ash) were expressed as per unit mass and they sum up to a constant value, they were not independent of one another. In other words, these compositional data were under the constant-sum constraint. In contrast, energy was not calculated as a part of compositional data, and the factors that had relatively small content levels (i.e., sodium, potassium, and cesium) were treated as noncompositional data because their contributions to the whole composition were considered negligible. Thus, the five macronutrient-related factors were subjected to log-ratio analysis using centered log-ratio transformation (clr) [87,88,89] as follows: raw data for each locality were first divided by their geometric means, resulting in ratio transformed data, and they were further transformed into natural logarithmic values. We did not perform more complex treatments such as Dirichlet regression [90,91].

Since the transformation above was performed to make the data normally distributed, we performed Shapiro–Wilk test (*p* > 0.001) to check normality of these data. Normality was observed not only in the ratio transformed data and the clr data but also in the raw data at least in the three groups combined and in the Tohoku group (Table S10). Moreover, the raw data correlated well with the ratio transformed data and with the clr data (Table S11). On the basis of these results and for physiological simplicity, we considered it reasonable to treat the output factors to be independently normally distributed to understand linear relationships among them. This treatment was also reasonable for the present situation in which both compositional and noncompositional data were included together. Our standpoint was that as long as nature of compositional data was understood, statistical analyses based on the assumption of independent normality were reasonably insightful.

## 3. Results

### 3.1. Comparisons of Nutrient Factors among the Tohoku, Niigata, and Kyushu Groups

**Figure 3:**
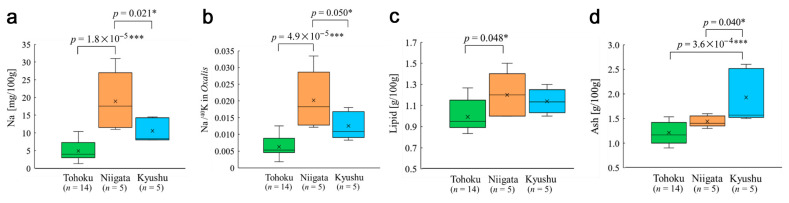
Comparisons among three locality groups using the Tukey–Kramer test. Only statistically significant pairs are indicated with *p*-values. *: *p* < 0.05, ***: *p* < 0.001. (**a**) Sodium content. (**b**) Na/^40^K ratio. (**c**) Lipid content. (**d**) Ash content.

When the levels of the nutrient factors were compared among the three different locality groups, statistically significant differences in the sodium contents were observed between the Tohoku and Niigata groups (*p* = 1.8 × 10^−5^) and between the Niigata and Kyushu groups (*p* = 0.021) (Figure 3a). Notably, the former was highly significant. The sodium content of the Tohoku group was also lower than that of the Kyushu group, although this difference was not significant (*p* = 0.061).

Although not significant, the potassium content was close to the significance level between the Tohoku and Niigata groups (*p* = 0.052) (not shown). Furthermore, elimination of a potential outlier, the largest value from Yamagata (Table S2), made the difference in the potassium content significant between the Tohoku and Niigata groups (*p* = 0.0019) and between the Tohoku and Kyushu groups (*p* = 0.047) (not shown). When the Na/^40^K ratio was compared as a cationic balance indicator in the plant, significant *p*-values were obtained between the Tohoku and Niigata groups (*p* = 4.9 × 10^−5^) and between the Niigata and Kyushu groups (*p* = 0.050) (Figure 3b), which was similar to the results of the sodium content (Figure 3a). The Na/^40^K ratio of the Tohoku group was also lower than that of the Kyushu group, although this difference was not significant (*p* = 0.055).

The lipid content also showed a significant difference between the Tohoku and Niigata groups (*p* = 0.048), and the content between the Tohoku and Kyushu groups was not significant (*p* = 0.19) (Figure 3c). In addition, the ash content was significantly different between the Kyushu and Tohoku groups (*p* = 3.6 × 10^−4^) and between the Kyushu and Niigata groups (*p* = 0.040) (Figure 3d). Other nutrient factors did not show significant differences among these three groups (not shown).

### 3.2. Radioactive Factors and Nutrients: Sodium and Lipid Contents

The lower levels of a few nutrient contents in the Tohoku group in the previous section could imply a biological influence of radioactive pollution in the Tohoku group. Here, we examined whether the nutrient factors (the output variables) were correlated with the radioactive factors (the input variables) in the Tohoku group (Table S6). The radioactivity concentration of cesium (^137^Cs) and the ground radiation dose in the Niigata and Kyushu groups were below the detection limits in our analytical methods here, which made statistical analyses in this section possible only in the Tohoku group.

**Figure 4:**
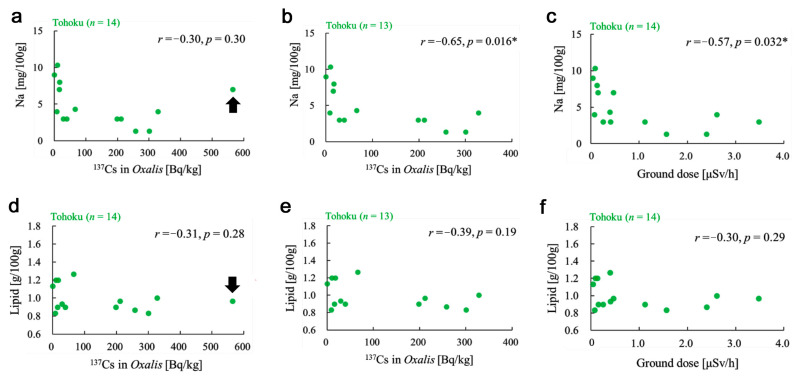
Scatter plots of the sodium and lipid contents in response to the two radioactive factors (radioactivity concentration of ^137^Cs and ground dose) in the Tohoku group. Pearson correlation coefficients *r* with their associated *p*-values are shown. *: *p* < 0.05. (**a**) Sodium versus ^137^Cs using all 14 locality samples. A single Tomioka-1 sample is indicated by an arrow and is excluded in (b). (**b**) Sodium versus ^137^Cs using 13 locality samples. (**c**) Sodium versus ground dose. (**d**) Lipid versus ^137^Cs using all 14 locality samples. A single Tomioka-1 sample is indicated by an arrow and is excluded in (e). (**e**) Lipid versus^137^Cs using 13 locality samples. (**f**) Lipid versus ground dose.

The correlation between the radioactivity concentration of cesium and the sodium content in the plant appeared to be low and not significant when using all 14 locality samples (*r* = −0.30, *p* = 0.30) (Figure 4a). However, the scatter plot indicated that there may be an outlier (i.e., the highest ^137^Cs point of the Tomioka-1 sample); this point was isolated from the rest. The ^137^Cs level of Tomioka-1, which is one of the geologically closest localities to the Fukushima Dai-ichi Nuclear Power Plant examined here, was very high compared to the others, justifying this exclusion. When it was excluded, a reasonably high negative correlation was obtained with a reasonably significant *p*-value (*r* = −0.65, *p* = 0.016) (Figure 4b). Additionally, the sodium content was negatively correlated with the ground radiation dose of the plant sampling site (*r* = −0.57, *p* = 0.032) (Figure 4c). In contrast, the lipid content did not show a significant correlation with the radioactivity concentrations of ^137^Cs using all 14 locality samples (Figure 4d). The correlation was still not significant after the exclusion of an outlier (i.e., Tomioka-1) (Figure 4e). The correlation between the lipid content and the ground dose was also not significant (Figure 4f).

**Figure 5:**
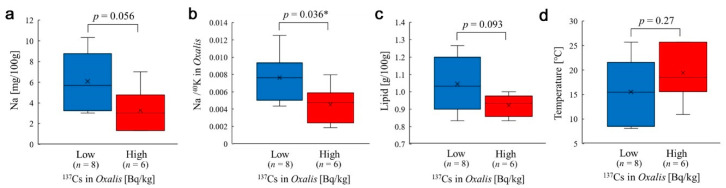
Box plots comparing the high-dose and low-dose subgroups of the radioactivity concentration of ^137^Cs in the Tohoku group using Welch’s *t*-test. *: *p* < 0.05. (**a**) Sodium content. (**b**) Na/^40^K ratio. (**c**) Lipid content. (**d**) Temperature.

Above, we assumed linear relationships between two variables. An alternative interpretation of the distribution of the radioactivity concentration of ^137^Cs was that the distribution could be divided into two parts by the large gap into the high-dose and low-dose subgroups; this gap was between Ryozen (66.16 Bq/kg) and Tomioka-2 (198.08 Bq/kg). The ^137^Cs radioactivity concentrations between these subgroups were significantly different (*p* = 0.0032). When comparing these subgroups, the sodium content was not significantly different (*p* = 0.056) (Figure 5a), but the Na/^40^K ratio was significantly different (*p* = 0.036) (Figure 5b). The lipid content did not show a significant difference between the high-dose and low-dose subgroups (*p* = 0.093) (Figure 5c). No other nutrient factors showed significant differences between the high-dose and low-dose subgroups (not shown). To examine whether temperature may have contributed to these differences, temperature distributions were compared between these high-dose and low-dose subgroups; no statistically significant difference was obtained (Figure 5d).

In summary, the sodium content was likely influenced by radioactive factors associated with the Fukushima nuclear accident. Changes in the lipid content in response to these radioactive factors were weak, if any. Other nutrient factors were not correlated with these two radioactive factors.

### 3.3. Correlations of the Sodium Content with Other Nutrient Contents

**Figure 6:**
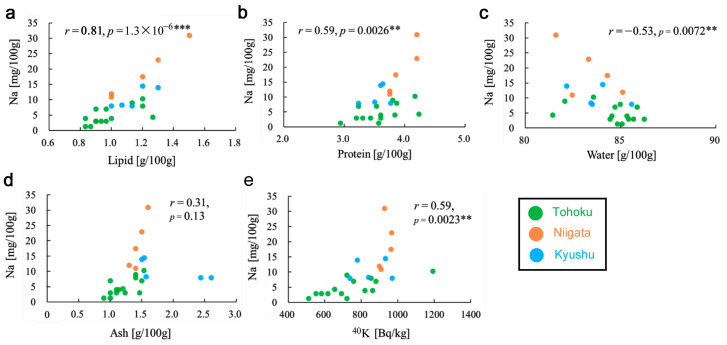
Scatter plots and correlation coefficients between the sodium content and other nutrient contents. **: *p* < 0.01, ***: *p* < 0.001. (**a**) Lipid content. (**b**) Protein content. (**c**) Water content. (**d**) Ash content. (**e**) ^40^K radioactivity concentration.

Thus far, it is possible that the sodium content in the *Oxalis* plants changed in response to radioactive pollution. To examine whether the sodium content may interact with other nutrients, correlations between the sodium content and other nutrient factors were examined using all samples from the three locality groups (Table S5). The sodium content was positively correlated with the lipid content (*r* = 0.81, *p* = 1.3 × 10^−6^) (Figure 6a), protein content (*r* = 0.59, *p* = 0.0026) (Figure 6b), and energy (*r* = 0.52, *p* = 0.0090) (not shown). The sodium content was negatively correlated with the water content (*r* = −0.53, *p* = 0.0072) (Figure 6c). The sodium content was not correlated with the carbohydrate content (*r* = 0.25, *p* = 0.23) (not shown) or the ash content (*r* = 0.31, *p* = 0.13) (Figure 6d) but was correlated with the ^40^K radioactivity concentration (*r* = 0.59, *p* = 0.0023) (Figure 6e). It appeared that sodium might be related to various biosynthetic and physiological pathways in this plant.

### 3.4. Coefficient of Variation among the Nutrient Factors

**Figure 7:**
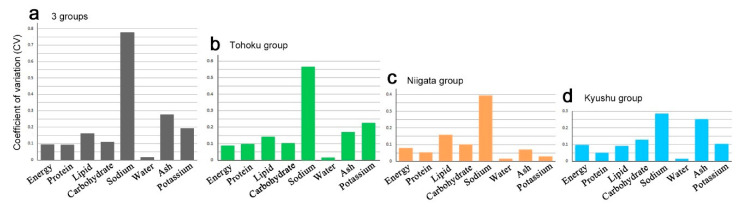
Coefficient of variation (CV) among the nutrient factors. (**a**) Three groups combined. (**b**) Tohoku group. (**c**) Niigata group. (**d**) Kyushu group.

To understand variability of the nutrient factors in the plant, we calculated the coefficient of variation (CV), a unitless value for relative variability (Table S9). Comparison of the CV among the nutrient factors indicated that the sodium content was by far the most variable factor when all samples from the three groups were used (Figure 7a). In contrast, the water content was the least variable factor. Other nutrient factors were found between the sodium and water contents.

The overall CV trend was also observed in the Tohoku group (Figure 7b), the Niigata group (Figure 7c), and the Kyushu group (Figure 7d), but the CV of the sodium content in the Tohoku group was largest among the three groups. The CV of the sodium content was even larger when all samples from the three groups were used (Figure 7a), reflecting distribution range differences of the sodium content among the three groups. The CV of the potassium content varied among the three groups and was relatively large in the Tohoku group, which was consistent with the sodium-potassium positive correlation (Figure 6e).

### 3.5. Temperature Effects on Nutrient Contents

**Figure 8:**
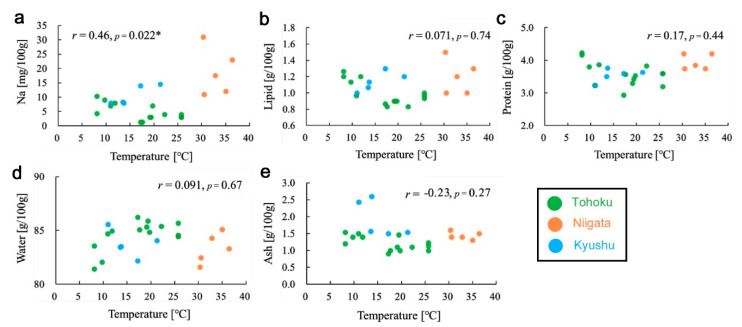
Scatter plots and correlation coefficients between temperature and nutrient contents. *: *p* < 0.05. (**a**) Sodium content. (**b**) Lipid content. (**c**) Protein content. (**d**) Water content. (**e**) Ash content.

Since temperature may be one of the important environmental factors that could affect plant physiology, correlations were examined between nutrient contents and temperature at the sampling site using all samples from the three groups (Table S5). Sodium content was significantly positively correlated with temperature (*r* = 0.46, *p* = 0.022) (Figure 8a). In contrast, the lipid content (*r* = 0.071, *p* = 0.74) (Figure 8b), protein content (*r* = 0.17, *p* = 0.44) (Figure 8c), water content (*r* = 0.099, *p* = 0.67) (Figure 8d), ash content (*r* = −0.23, *p* = 0.27) (Figure 8e), carbohydrate content (*r* = −0.093, *p* = 0.67) (not shown), and energy (*r* = −0.021, *p* = 0.92) (not shown) showed very low correlation coefficients with relatively high *p*-values.

When only samples from the Tohoku group were subjected to correlation analyses, the protein content (*r* = −0.48, *p* = 0.081) and the ^40^K radioactivity concentration (*r* = −0.37, *p* = 0.19) were not significant. However, the water content (*r* = 0.71, *p* = 0.0044) and ash content (*r* = −0.57, *p* = 0.034) were significant, contrasting with the previous cases when all samples from the three groups were used, but the signs of the coefficients were consistent. In contrast, the sodium content (*r* = −0.63, *p* = 0.015) and the lipid content (*r* = −0.69, *p* = 0.0062) showed significant negative correlation coefficients with temperature when using only the Tohoku group, which contrasted with the positive correlation coefficients when using all samples from the three groups. That is, the signs of coefficients changed between the two analyses with both significant *p*-values, only in the sodium content.

**Figure 9:**
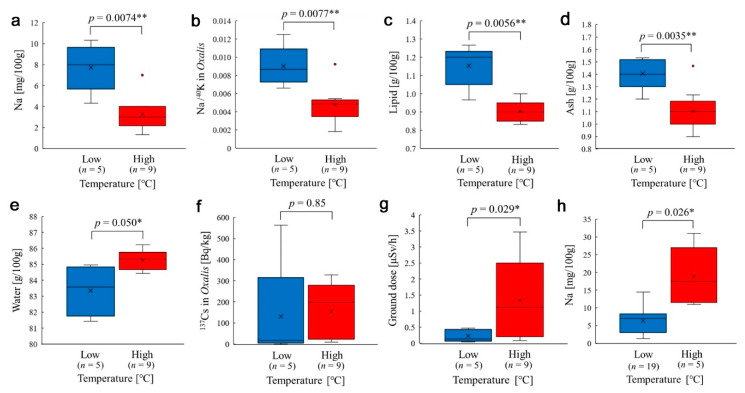
Box plots comparing high-temperature and low-temperature subgroups in the Tohoku group (except h) using Welch’s *t*-test. *: *p* < 0.05, **: *p* < 0.01. (**a**) Sodium content. (**b**) Na/^40^K ratio. (**c**) Lipid content. (**d**) Ash content. (**e**) Water content. (**f**) Radioactivity concentration of ^137^Cs in *Oxalis*. (**g**) Ground dose. (**h**) Sodium content using all sample data from the three groups.

To further understand the possible temperature effect on the nutrient factors in the Tohoku group, comparisons were made between the high-temperature and low-temperature subgroups within the Tohoku group. These subgroups were divided at the largest gap between Iwaki-1 (11.8 °C) and Iitate-1 (17.2 °C). The temperatures between these subgroups were significantly different (*p* = 2.6 × 10^−6^). We found that between the high-temperature and low-temperature subgroups, the sodium content (*p* = 0.0074) (Figure 9a), Na/^40^K ratio (*p* = 0.0077) (Figure 9b), lipid content (*p* = 0.0056) (Figure 9c), ash content (*p* = 0.0035) (Figure 9d), and water content (*p* = 0.050) (Figure 9e) were all significantly different. In these cases, the contents were lower in the high-temperature subgroup than in the low-temperature subgroup, with the exception of the water content.

Similar comparisons were made between the high-temperature and low-temperature subgroups in terms of the radioactivity concentration of ^137^Cs in *Oxalis* leaves and the ground radiation dose. The former was not significant (*p* = 0.85) (Figure 9f), but the latter showed a significant difference (*p* = 0.029) (Figure 9g), suggesting that temperature and the ground dose may be related geographically in the Tohoku district.

For comparison with the sodium content distributions in the high-temperature and low-temperature subgroups in the Tohoku group (Figure 9a), the sodium content distributions using all samples from the three groups were examined (Figure 9h). In this case, the gap between the subgroups was located between Namie-2 in the Tohoku group (25.7° C) and Yahiko in the Niigata group (30.3 °C). The temperatures between these subgroups were significantly different (*p* = 1.9 × 10^−7^). As expected, the high-temperature and low-temperature subgroups were significantly different (*p* = 0.026), confirming the different trend from the result of the Tohoku group. That is, the temperature–sodium relationship might have been reversed in the Tohoku group.

### 3.6. Additional Analyses: Input Variables and Seasonal Comparisons

In this study, we used two radiation-related variables, the ^137^Cs radioactivity concentration in *Oxalis* leaves and the ground radiation dose, as input variables to understand the possible nutrient differences. In addition, temperature was used as an input variable. To aid interpretations of the results obtained thus far, we examined relationships among these input variables (Supplementary Results; Figures S1 and S2).

Additionally, in this study, we focused on temperature, assuming that seasonal features could be reduced to temperature. Here, to examine potential seasonal features, we compared (1) the Tohoku and Kyushu groups both collected in winter, (2) the Tohoku and Niigata groups both collected in summer, and (3) the summer and winter samples in the Tohoku group (Supplementary Results; Figures S3–S5).

## 4. Discussion

### 4.1. Physiological Changes in Nutrient Contents in the Host Plant

The present study examined the feasibility of the field-effect hypothesis stating that the creeping wood sorrel *Oxalis corniculata*, the host plant of the pale grass blue butterfly, may change its nutrient contents for butterfly larvae in response to radioactive pollution caused by the Fukushima nuclear accident. To the best of our knowledge, this approach to nuclear pollution issues, which is based on nutrient analyses of plants from the perspective of field effects on animals, is a novel attempt. The results of the present study showed that a decrease in the sodium content might have occurred in response to radioactive pollution in the plants in the Tohoku group, which is consistent with the field-effect hypothesis. Further studies are required to prove this hypothesis.

From the view of environmental radioprotection, discrepancies in the biological effects of radioactive pollution between dosimetric data from controlled laboratory exposure experiments and those from field work have been recognized, and efforts to fill these gaps are in progress [92,93,94]. Higher sensitivities of organisms to radiation exposure in wildlife than in the laboratory have often been attributed to nonradiological environmental (both abiotic and biotic) factors. In the case of the present study on the creeping wood sorrel and the pale grass blue butterfly, the “radiological sensitivity” of the butterfly may not differ much between the laboratory and field conditions, but the “ecological toxicity” of radionuclides to the butterfly is amplified by the plant in the field as the nutrient changes. Although this may not be a comprehensive explanation, ecological field effects of radiation are to be considered in discussing radiological environmental protection in general. Other types of effects are also theoretically possible; for example, contributions of particulate matter from the accident as air pollutants and of barium (^137^Ba) as a radioactive decay product of cesium (^137^Cs) may be in effect.

It is important to recognize that for plants, radiation-induced chemical changes in leaves are the “direct” or “primary” effects of radiation exposure rather than the “indirect” or “secondary” effects that occur in herbivorous insects. Most plants do not seem to change any morphology at the low-dose exposure levels in Fukushima, but this could cause serious ecological consequences if all plants in the polluted area were stressed simultaneously by radioactive pollution and changed their nutritional or other chemical factors.

### 4.2. Status of Kyushu and Tomioka-1

Overall, the results of the present study clearly showed that among the nutrient factors (i.e., the “output” variables), sodium was the most significant response candidate to the two radioactive “input” variables in the Tohoku group. The sodium content was highly different between the Tohoku and Niigata groups (Figure 3a). Although the lipid content also showed a significant difference between the Tohoku and Niigata groups (Figure 3c), its *p*-value was relatively large, and indeed, the lipid content was unlikely to respond to the radioactivity factors. However, the differences in the sodium content (*p* = 0.061) and lipid content (*p* = 0.19) between the Tohoku and Kyushu groups were not significant. We believe that the Niigata group serves as a better control group than the Kyushu group because the Tohoku and Niigata districts are located at similar latitudes. An increase in sample size could make the difference in the sodium content between the Tohoku and Kyushu groups significant, but there may be a genetic difference between these groups that could mask a difference in the sodium content between them.

One outlier (the Tomioka-1 sample) was excluded in the correlation analyses. This sample had a relatively low ground radiation dose but a very high ^137^Cs radioactivity concentration (Supplementary Results; Figure S1a). We believe that the exclusion of this data point can be justified based on the unusual values that were far from the nearest values. Most likely, these unusual values came from extensive decontamination efforts in this area; for example, surface soil has been removed, as reflected in the low ground radiation dose, but the plant might have absorbed or adsorbed a high dose of ^137^Cs. Indeed, when the Tomioka-1 sample was excluded, the ^137^Cs radioactivity concentration appeared to be saturated at approximately 300 Bq/kg (Supplementary Results; Figure S1b). It is likely that the Tomioka-1 samples adsorbed ^137^Cs on the surface of leaves.

### 4.3. Temperature Effect in the Tohoku Group

Since temperature generally affects plant physiology, we examined the possible temperature effect on *Oxalis corniculata*. When all sample data from three locality groups were used, no correlation of nutrients with temperature was found except for sodium, in which case a higher temperature was associated with a higher sodium content. However, when only samples from the Tohoku group were used, negative correlations were observed in the sodium content and in some other nutrient contents. That is, in the Tohoku group, the sodium content was negatively influenced both by temperature and pollution level (^137^Cs in leaves and ground dose). This contrasting result was also clear when the high- and low-temperature subgroups were compared using only the Tohoku samples (Figure 9a) and using all samples from the three groups (Figure 9h). A possible interpretation is that the normal temperature–sodium relationship was reversed in the Tohoku district due to radiation stress. Furthermore, the negative response of sodium to both radiation and temperature in the Tohoku group may suggest a synergistic stress effect of radioactive pollution and high temperature.

However, lipids and ash also appeared to decrease in response to high temperature in the Tohoku group, although these results do not necessarily mean a nonspecific nature of the sodium content decrease. A relatively large part of the ash content may indeed be composed of sodium, and lipids may be considered another candidate that responds to temperature but not to radioactivity factors.

The sodium–cesium negative correlation (Figure 4b) is a critical result in the present study. This correlation may be remarkable, considering that the data for this analysis were obtained under two different temperatures in different seasons (summer (July 2016) and winter (November 2020)) and that the sodium content was likely negatively affected by temperature in the Tohoku group (Figure 9a). In the scatter plot (Figure 4b), there are four data points showing low cesium but relatively low sodium; Sendai-2, Ryozen, Minamisoma, and Soma. They do not seem to fit the liner trend well. Among these four data points, only Ryozen is a winter locality. Other three points are summer localities, which may be a reason for their relatively low sodium. Sendai-2 (4 mg), which is a summer locality, may be compared with Sendai-1 (9 mg), which is a winter locality. Expectedly, the sodium content of Sendai 2 is lower than that of Sendai-1. The relatively low sodium content in the plant in summer may affect the population of the pale grass blue butterfly more severely than in winter, simply because the butterfly is more active in summer than in winter.

The sodium content was also negatively correlated with the ground radiation dose (Figure 4c), and the ground radiation dose was significantly larger in the high-temperature subgroup than in the low-temperature subgroup (Figure 9g). These results are understandable because the Fukushima Dai-ichi Nuclear Power Plant is located at the seashore, which is the warmest place in Fukushima. The fact that plant sampling in high-dose localities was carried out in summer when temperatures were relatively high might also have resulted in this correlation between the temperature and ground radiation dose.

We also investigated potential seasonal differences that might not be discovered in just probing temperature (Supplementary Results; Figures S3–S5). All nutrient factors except the carbohydrate content were significantly different between the Tohoku and Niigata groups collected in summer, which could be due to differences in genetic information, radioactive pollution, or simply temperature in summer. These differences were not found except the sodium content when the Tohoku group collected in winter was compared with the Niigata group collected in summer. Thus, only sodium was significantly different irrespective of seasons. A possible interpretation is that sodium reflects regional differences more than other nutrient factors. A regional factor that made sodium different might be radioactive pollution in the Tohoku district.

### 4.4. Possible Function of Sodium and Radiation-Induced Stress Response in the Plant

In this study, somewhat unexpectedly, sodium showed correlations with other nutrient factors, such as the lipid and protein contents, when all samples from the three groups were used. Thus, there is a possibility that, in *Oxalis corniculata*, sodium may play a regulatory role in adjusting the metabolic pathways for lipids and proteins in response to radiation stress. The analytical method for the protein contents used in this study is unlikely to be sensitive enough to detect upregulation or downregulation of other nitrogen-containing molecules such as amino acids or their related chemicals such as proline, betaines, and polyamines known as compatible solutes in osmotic adjustment upon abiotic stress [95].

Sodium is known to function as a substitute for potassium in plants [96,97,98,99], but in this study, sodium was positively (but not negatively) correlated with potassium (as well as with ^137^Cs). The sodium content and the Na/^40^K ratio showed similar results with minor *p*-value differences (Figure 3a,b, Figure 5a,b and Figure 9a,b). It appears that sodium and potassium change together proportionally, as seen in the scatter plot (Figure 6e). It should be noted that the potassium content was close to the significance level between the Tohoku and Niigata groups (*p* = 0.052). These results may suggest the importance of the sodium/potassium ratio for this plant. However, there is a possibility that sodium absorption may occur as an “error” of potassium absorption.

The sodium content was the one that changes most in the plant among the nutrient factors examined here, as indicated by the largest CV. This was true not only in the Tohoku group but also in the Niigata and Kyushu groups. The potential implication is that sodium may be either a key player for physiological changes or just an opportunistic visitor that does not involve any physiological function. We believe that the former is likely, based on the present finding that the sodium content was correlated with many other factors. There is a possibility that the sodium content may simply reflect soil composition. Regrettably, we did not analyze soil composition, but we believe that a contribution of soil composition to the sodium content is unlikely to be large, considering that the sodium content is negatively correlated with the radiation variables.

In contrast, the water content did not change much, as indicated by the smallest CV, and it was nearly insensitive to temperature (8.1–36 °C) in the present study. This is probably because a constant level of the water content is very important for plant homeostasis. Although there is a reasonable negative correlation between the sodium and water contents, a contribution of the water content to the sodium content is unlikely to be large.

Other nutrient factors had the CV values between the sodium and water contents (Figure 7). This overall CV trend was largely expected; the CV always becomes smaller in the factors that have inherently large content values, because there is little room for their variability in the first place. However, the factors that have inherently small content values do not necessarily have high CV values. In this sense, the high CV value of the sodium content was not surprising but important, because it may potentially reflect not only the small content value of sodium but also physiological function of sodium in the plant.

We did not detect a correlation between potassium and cesium in the present study. This result is not consistent with a previous study in which the ^137^Cs and ^40^K levels in several weed plants were correlated well in 2014–2016 in radioactively polluted areas [55]. However, it has also been reported that a fraction of ^137^Cs in soil that can be absorbed by plants appears to be largely constant after 2014 [54] and that cesium shows a different distribution pattern from potassium in plants [56]. Considering the importance of potassium in the stress response [100], the relationships among sodium, potassium, and cesium need to be clarified in the future.

The sodium content in the whole plant of *Oxalis corniculata* in India has been reported to be 22.5 ± 2.4 mg/100 g [101]. This value is similar to the value of Murakami City in the Niigata group, which was 23 mg/100 g, although only leaves were used in the present study. In the present study, the sodium content varied from 1 mg/100 g (Iitate Village, Fukushima Prefecture) to 31 mg/100 g (Yahiko Village, Niigata Prefecture). The changes in the sodium content in this range may not be physiologically significant for the survival of this plant. The creeping wood sorrel can thrive along seashores, which implies that this plant is tolerant of sodium to some extent. At least in a macroscopic view, the plant did not show any morphological abnormalities and looked robust in the contaminated field; we did not observe noticeable levels of leaf necrosis, chlorosis, or other abnormalities. For many plants, sodium is an important beneficial or functional element but is not an essential element for survival except for some C_4_ and CAM plants [96,97,98,99]. *Oxalis corniculata* is likely a C_3_ plant, and it is believed that C_3_ plants do not require sodium for its physiological functions [96,97,98,99].

When plants are exposed to various kinds of abiotic stress, at least a few different pathways may be activated, and one of them is a pathway mediated by reactive oxygen species (ROS), such as H_2_O_2_ [99,102,103,104,105,106]. In this case, ROS are produced from mitochondria, chloroplasts, and peroxisomes and function as signaling molecules to change various aspects of plants, including sodium/potassium homeostasis and gene expression patterns [99,102,103,104,105,106]. Whether plant responses to radiation stress involve the similar production of ROS as a signaling molecule that results in changes in the sodium content is unknown, but because ROS are passively produced in plants due to ionizing effect of radiation when exposed, a systematic production and signaling of ROS may be obscured by the high background noise of ROS that are randomly produced by ionizing radiation. Alternatively, the ROS induced by ionizing radiation may function as signaling molecules to efficiently cope with radiation stress. Inevitably, antioxidant production may accompany the ROS production. Ascorbic acid (vitamin C) is known to be produced more when potassium is lower in leaf vegetables [107,108]. Considering that sodium and potassium were positively correlated and that sodium responded to the radioactive factors in the present study, it is possible that the *Oxalis* plant may lower the sodium content to increase antioxidants and to cope with the high background noise of ROS when exposed to ionizing radiation.

### 4.5. Herbivorous Insects and Their Sodium Requirement

Herbivorous land vertebrates (mostly mammals) require sodium not only from plants that they consume but also from additional sources such as rock salts for their survival [109,110,111]. This is because most plants that they consume do not contain enough sodium to sustain animals’ physiological requirements due to differences in ion usage between plants and animals. Herbivorous insects such as butterflies have to obtain sodium exclusively from host plants without external sources, at least during the larval stage. Therefore, the sodium content of the host plant may be a critical factor affecting the survival of herbivorous insects but not the survival of plants. If so, this plant response to radiation stress would be functionally suitable for the plants to protect themselves from being eaten by herbivorous insects under stressful conditions.

Plants have many defense mechanisms. For example, upon insect attack, plant leaves synthesize a small peptide, systemin, as a systemic wound-signaling molecule, which then induces proteinase inhibitors that could inhibit protein digestion when ingested by larvae [112,113]. It is known that when plants are stressed, they produce many chemical factors (i.e., allomones) and change nutrient contents [114]. Undoubtedly, insects that depend on stressed plants are less robust; they are not killed immediately, but they show growth retardation and become susceptible to diseases and other environmental factors [115].

Indeed, the importance of sodium in butterflies is known. The consumption level of sodium at the larval stage affects the development of the neuromuscular system of adult butterflies [116], although foraging behaviors of adults and larvae for sodium-rich diets have not been observed [117]. The puddling behavior of adults is basically to obtain salts, especially sodium, which is positively related to reproductive and mating success [118,119,120]. In the case of amino acids, the nutritional quality of the diet at the larval stage determines the preference of adults for nectar containing amino acids in females but not in males in a nymphalid butterfly [121]. More generally, the nutritional variability of plants is likely a key to the status of plants as diet suppliers for insect herbivores [122].

In the highly polluted area of Chernobyl, population sizes of many animal taxa were reduced, including birds, bees, butterflies, grasshoppers, dragonflies, spiders, and mammals [36,37]. Additionally, there are many observations that suggest potential changes in ecological community in response to radioactive pollution at low dose levels [123,124,125], which are potentially through the field effects. In contrast, in Fukushima, where the pollution levels are lower than those in Chernobyl, population sizes of birds, butterflies, and cicadas were reduced, while those of dragonflies, grasshoppers, bees, and spiders were not reduced significantly [36,37]. Butterflies and cicadas are herbivorous or phytosuccivorous insects whose larvae or nymphs totally depend on the host plants for their survival. The larvae or nymphs of these insects cannot move much and consume leaves or tree sap of a particular batch of grasses or trees. When the host plant is under radiation stress, these insects will be affected if the nutritional content of the host plant changes. A decrease in bird populations could be attributed to a prompt decrease in lepidopteran populations because lepidopteran insects are important foods for birds.

In contrast, grasshoppers can move and consume various plants, making them less likely to become nutritionally deficient. Dragonflies, bees, and spiders were carnivorous or omnivorous, not depending on a single plant. Monophagous herbivorous insects may be the most vulnerable in the field, as seen in Fukushima, and when the pollution level is high, omnivorous and carnivorous insects and spiders may also be affected, as seen in Chernobyl. Consistent with this argument, a group of insects that showed striking morphological abnormalities after the Fukushima nuclear accident are gall-forming aphids, whose life history is dependent on the host plant [39,40].

### 4.6. Transgenerational Effects of Ingesting Radioactively Polluted Diets

Our previous field sampling study on the pale grass blue butterfly in 2016 (5.5 years after the accident) showed that butterflies in highly contaminated areas still exhibited a small level of morphological abnormalities [76]. These abnormalities have been suggested to originate both from the initial high-dose exposures immediately after the accident and from subsequent transgenerational effects [76]. The former is a genetic effect due to DNA damage, whereas the latter is a physiological effect. In the latter case, the butterfly larvae are affected by ingesting the contaminated host plant. The larvae are affected “directly” by radiation exposure through long-term ingestion of contaminated leaves, but simultaneously, they are also affected “indirectly” through changes in plant biochemicals, as suggested in the present study. Indeed, adverse effects of ingesting radioactively contaminated host plants in pale grass blue butterflies appear to be transgenerational [38,58,59,60,61,62,63,64,73], but the effects can be reversed when a noncontaminated diet is ingested [61]. The ingestion of radioactive materials likely causes a decrease in granulocytes in the hemolymph of the cabbage white butterfly, which then may cause decreases in the pupal eclosion rate, adult achievement rate, and total normality rate [45]. These adverse effects are probably mediated at least partially not by a direct radiation effect on the larvae but by an indirect field effect through changes in plant nutrients or other biochemicals.

### 4.7. Essential Nutrients and Secondary Metabolites

This study showed the possibility of the field effect of radioactive pollution on herbivorous insects through nutrient changes in their host plant. Theoretically, biochemical changes in the host plant are not restricted to sodium. Any decreases in essential nutrients such as vitamins, amino acids, and minerals may also cause deficiency diseases that result in larval growth retardation and developmental malformation. In addition, an increase in secondary metabolites such as insect repellents and toxicants that are upregulated under stress conditions may cause the field effect. To investigate this potential mechanism, metabolomics and ionomics of leaves and rearing experiments of larvae using artificial diets with various sodium levels are expected in the future. Furthermore, the ecological field effect should be considered one of many types of field effects theoretically possible in the field [66,71]. Other potential field effects of radioactive pollution include physical and immunological effects of particulate matter, chemical toxic effects of metals, and synergistic effects of environmental stress such as climate changes and agrochemicals [66,71].

## 5. Conclusions

In this study, we showed low sodium contents in the creeping wood sorrel in radioactively contaminated areas. The sodium content in this plant was likely affected by radiation exposure in the field, although the plant itself did not seem to show any morphological abnormalities following radiation exposure. Changes in the sodium content in the host plant may be one of the causes of high mortality and abnormality rates previously detected in the pale grass blue butterfly in Fukushima, although other possibilities should also be investigated. This study suggests the possibility of ecological field effects of radioactive pollution on wild animals in Fukushima and possibly in other radioactively polluted areas.

## Data Availability

The data presented in this study are available in Supplementary Materials.

## Supplementary Materials

The following are available online at https://www.mdpi.com/2075-4450/12/2/149/s1, This paper is accompanied by Supplementary Materials in two PDF files (Supplementary Results containing Figures S1–S5 and Supplementary Tables containing Tables S1–S8). Figure S1: Scatter plots and correlation coefficients between the ^137^Cs radioactivity concentration and the ground radiation dose in the Tohoku group. Figure S2: Scatter plots and correlation coefficients between temperature and the ^137^Cs radioactivity concentration or the ground radiation dose in the Tohoku group. Figure S3: Comparisons of the nutrient contents between the Tohoku and Niigata groups both collected in summer using Welch’s *t*-test. Figure S4: Comparisons of the sodium content between the Tohoku group collected in winter and the Niigata group collected in summer using Welch’s *t*-test. Figure S5: Comparisons of temperature between the Tohoku and Niigata groups using Welch’s *t*-test. Table S1: Sampling information and environmental data, Table S2: Nutrient contents and ^40^K and ^137^Cs radioactivity concentrations in *Oxalis* leaves in the Tohoku group, Table S3: Nutrient contents and ^40^K and ^137^Cs radioactivity concentrations in *Oxalis* leaves in the Niigata group, Table S4: Nutrient contents and ^40^K and ^137^Cs radioactivity concentrations in *Oxalis* leaves in the Kyushu group, Table S5: Pearson correlation coefficients and their associated *p*-values among nutrient contents, ^40^K and ^137^Cs radioactivity concentrations in *Oxalis* leaves, temperature, and ground radiation dose in three groups combined (*n* = 24), Table S6: Pearson correlation coefficients and their associated *p*-values among nutrient contents, ^40^K and ^137^Cs radioactivity concentrations in *Oxalis* leaves, temperature, and ground radiation dose in the Tohoku group (*n* = 14), Table S7: Pearson correlation coefficients and their associated *p*-values among nutrient contents, ^40^K radioactivity concentration in *Oxalis* leaves, temperature, and ground radiation dose in the Niigata group (*n* = 5), Table S8: Pearson correlation coefficients and their associated *p*-values among nutrient contents, ^40^K radioactivity concentration in *Oxalis* leaves, temperature, and ground radiation dose in the Kyushu group (*n* = 5). Table S9: Coefficient of variation (CV) among nutrient contents and ^40^K radioactivity concentration in *Oxalis* leaves. Table S10: Shapiro–Wilk test for normality (*p*-values) using raw data, ratio transformed (rt) data, and centered log-ratio transformed (clr) data. Table S11: Pearson correlation coefficients and their associated *p*-values between raw data and ratio transformed (rt) data and between raw data and centered log-ratio transformed (clr) data.
