# Supplementary material for: Nutrient Imbalance of the Host Plant for Larvae of the Pale Grass Blue Butterfly May Mediate the Field Effect of Low-Dose Radiation Exposure in Fukushima: Dose-Dependent Changes in the Sodium Content
Source: Insects. 2021 Feb 9;12(2):149. doi: 10.3390/insects12020149 (PMC7916146; doi:10.3390/insects12020149)
Supplement: Supplementary file 1 [file insects-12-00149-s001.zip › Supplementary Results.pdf]

Nutrient imbalance of the host plant for larvae of the pale grass blue butterfly may mediate the field effect of low-dose radiation exposure in Fukushima: dose-dependent changes in the sodium content

Ko Sakauchi [1], Wataru Taira [1,2], Mariko Toki [1], Masakazu Tsuhako [1], Kazuo Umetsu [3], Joji M. Otaki [1,*]

[1] The BCPH Unit of Molecular Physiology, Department of Chemistry, Biology and Marine Science, Faculty of Science, University of the Ryukyus, Okinawa 903-0213, Japan
[2] Center for Research Advancement and Collaboration, University of the Ryukyus, Okinawa 903-0213, Japan
[3] Department of Forensic Medicine, Yamagata University School of Medicine, Yamagata 990-9585, Japan
* Correspondence: otaki@sci.u-ryukyu.ac.jp; Tel.: +81-98-895-8557

Supplementary Results

SR1.1. Relationships between Two Radiation-Related Variables

In this study, we used two radiation-related variables, the ^{137}Cs radioactivity concentration in *Oxalis* leaves and the ground radiation dose, as input variables to understand the possible effects of radioactive contamination on the plant. These two factors should be closely related because the plant likely absorbed ^{137}Cs from the ground and because ^{137}Cs was the major contaminant on the ground when the measurements were made. At first glance, these two variables were not correlated significantly when all Tohoku samples, including Tomioka-1, were used (r = 0.52, p = 0.058) (Figure S1a); however, as expected, when Tomioka-1 was excluded as an outlier sample, a high correlation coefficient with a low p-value was obtained (r = 0.86, p = 1.5 × 10^{-4}) (Figure S1b). According to this scatter plot, there appeared to be a saturation point near 300 Bq/kg. This high correlation coefficient suggests that the *Oxalis* plant indeed absorbed ^{137}Cs from the ground, causing physiological changes in the plant.

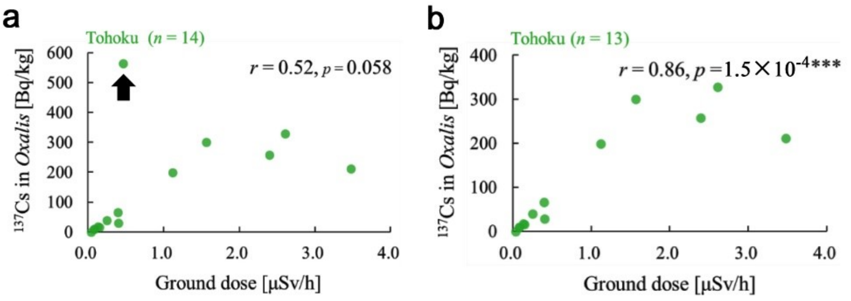

Figure S1. Scatter plots and correlation coefficients between the ^{137}Cs radioactivity concentration and the ground radiation dose in the Tohoku group. (**a**) Results using all 14 samples. An outlier sample (Tomioka-1) is indicated by an arrow. (**b**) Results after the exclusion of an outlier sample (Tomioka-1).

SR1.2. Relationships between Temperature and Two Radiation-Related Variables

In this study, we focused on temperature and two radiation-related variables as input variables to understand nutrient differences. However, since temperature appeared to be related to radiation-related factors, these relationships were further examined. Temperature and the ^{137}Cs radioactivity concentration in *Oxalis* leaves did not show a significant correlation using the Tohoku samples when Tomioka-1 was included (r = 0.083, p = 0.78) (Figure S2a) or when Tomioka-1 was excluded (r = 0.41, p = 0.16) (Figure S2b). However, temperature showed a significant correlation with the ground dose (r = 0.54, p = 0.049) (Figure S2c). This temperature-dose correlation was likely an unavoidable coincidence because the Fukushima nuclear power plant is located at the seashore, the warmest place in Fukushima.

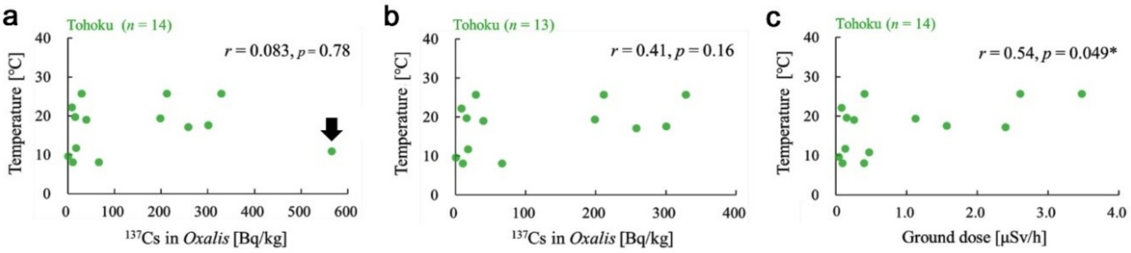

Figure S2. Scatter plots and correlation coefficients between temperature and the ^{137}Cs radioactivity concentration or the ground radiation dose in the Tohoku group. (**a**) Results using the entire 14 samples between temperature and ^{137}Cs radioactivity concentration. An outlier sample (Tomioka-1) is indicated by an arrow. (**b**) Results between temperature and ^{137}Cs radioactivity concentration after the exclusion of an outlier (Tomioka-1). (**c**) Results between temperature and ground radiation dose.

SR1.3. Seasonal Comparisons

In this study, we focused on temperature as an input variable to understand nutrient differences, assuming that seasonal features could be reduced to temperature. To examine potential seasonal similarities and differences that might not be reduced to temperature, here we compared (1) the Tohoku and Kyushu groups both collected in winter, (2) the Tohoku and Niigata groups both collected in summer, and (3) the summer and winter samples in the Tohoku group.

No significant difference was found in nutrient factors between the Tohoku and Kyushu groups both collected in winter (not shown).

In contrast, all nutrient factors except the carbohydrate content showed significant differences between the Tohoku and Niigata groups both collected in summer (Figures S3), suggesting that there might be regional (but not seasonal) differences. Interestingly, these significant differences disappeared except the sodium content (and the Na/^{40}K ratio) when the Niigata group collected in summer was compared with the Tohoku group collected in winter; only the sodium content (and the Na/^{40}K ratio) was significant (Figure S4). Although this section aimed at seasonal comparisons, it is to be noted that temperature difference was present between the Tohoku and Niigata groups both collected in summer (Figure S5a), and this difference was larger between the Tohoku group collected in winter and the Niigata group collected in summer (Figure S5b). It appears that the nutrient differences originate from regional and/or temperature differences and less likely from other seasonal features.

The summer and winter samples in the Tohoku group were the same as the temperature-based high and low subgroups, respectively (Figure 9).

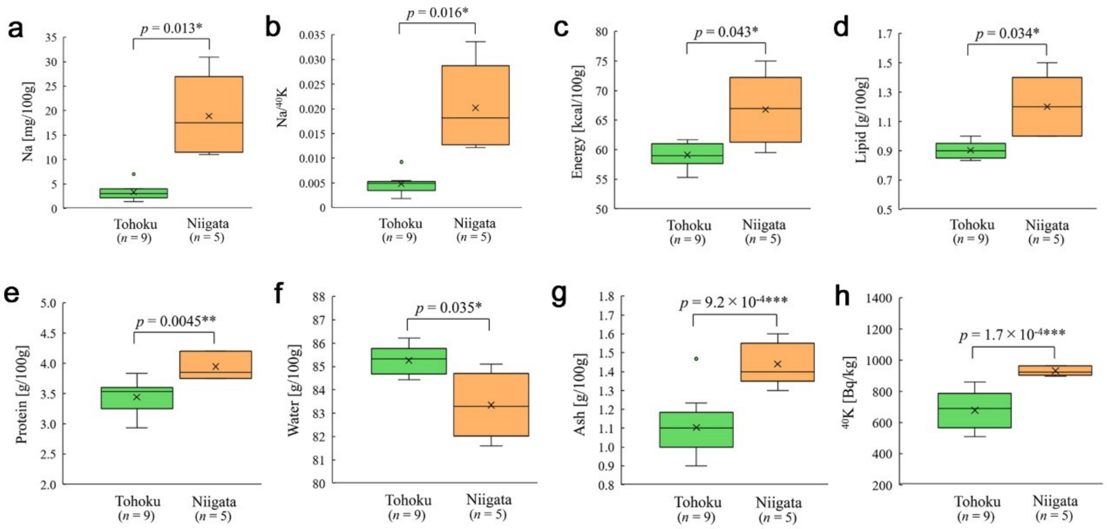

Figure S3. Comparisons of the nutrient contents between the Tohoku and Niigata groups both collected in summer using Welch's *t*-test. (**a**) Sodium content. (**b**) Na/^{40}K ratio. (**c**) Energy. (**d**) Lipid content. (**e**) Protein content. (**f**) Water content. (**g**) Ash content. (**h**) ^{40}K radioactivity concentration.

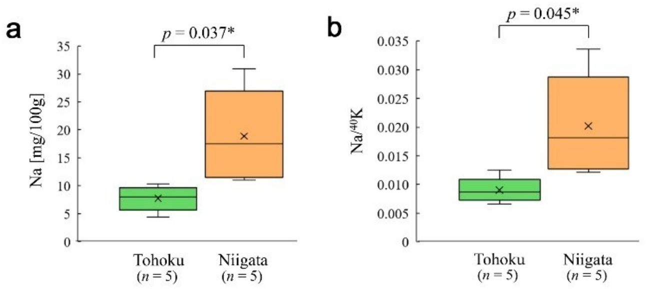

Figure S4. Comparisons of the sodium content between the Tohoku group collected in winter and the Niigata group collected in summer using Welch's *t*-test. (**a**) Sodium content. (**b**) Na/^{40}K ratio.

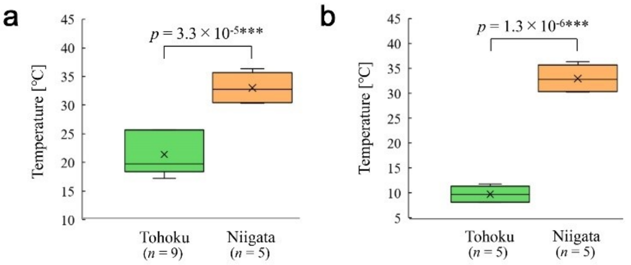

Figure S5. Comparisons of temperature between the Tohoku and Niigata groups using Welch's *t*-test. (**a**) Both groups collected in summer. (**b**) The Tohoku group collected in winter and the Niigata group collected in summer.
