# Supplementary material for: Nutrient Imbalance of the Host Plant for Larvae of the Pale Grass Blue Butterfly May Mediate the Field Effect of Low-Dose Radiation Exposure in Fukushima: Dose-Dependent Changes in the Sodium Content
Source: Insects. 2021 Feb 9;12(2):149. doi: 10.3390/insects12020149 (PMC7916146; doi:10.3390/insects12020149)
Supplement: Supplementary file 1 [file insects-12-00149-s001.zip › Supplementary Tables Jan 26 2021.pdf]

Table S1. Sampling information and environmental data.

Group	Prefecture	City/Town	Sampling date	Temperature [℃]	Ground dose [μSv/h]
Tohoku	Yamagata	Yamagata	2016/11/26	8.1	0.0880
	Miyagi	Sendai-1	2016/11/26	9.7	0.0400
		Sendai-2	2020/7/11	22.2	0.0760
	Fukushima	Ryozen	2016/11/27	8.1	0.3960
		Tomioka-1	2016/11/28	10.9	0.4680
		Iwaki-1	2016/11/28	11.8	0.1320
		Minamisoma	2020/7/11	25.7	0.4050
		Namie-1	2020/7/11	25.7	2.6060
		Soma	2020/7/12	19.1	0.2533
		Iitate-1	2020/7/12	17.2	2.3960
		Iitate-2	2020/7/12	17.6	1.5650
		Namie-2	2020/7/13	25.7	3.4757
		Tomioka-2	2020/7/13	19.4	1.1225
		Iwaki-2	2020/7/13	19.7	0.1500
Niigata	Niigata	Joetsu	2018/7/28	30.5	0.0433
		Kashiwazaki	2018/7/28	35.0	0.0300
		Murakami	2018/7/29	36.4	0.0700
		Niigata	2018/7/30	32.8	0.0533
		Yahiko	2018/7/30	30.3	0.0400
Kyushu	Kagoshima	Satsumasendai	2016/12/3	17.2	0.0520
		Izumi	2016/12/4	13.7	0.0600
	Kumamoto	Kumamoto	2016/12/4	11.0	0.0380
		Yatsushiro	2016/12/5	13.5	0.0540
	Okinawa	Nishihara-AM	2016/12/10	21.3	-
		Nishihara-PM	2016/12/10	22.6	0.0460

Note: Ground dose was calculated as the mean value of 3 to 7 spots. -: Not applicable.

Table S2. Nutrient contents and ^{40}K and ^{137}Cs radioactivity concentrations in *Oxalis* leaves in the Tohoku group.

City/Town	Replication, mean ± SD (*n*)	EN [kcal/100g]	PR [g/100g]	LI [g/100g]	CA [g/100g]	Na [mg/100g]	WA [g/100g]	AS [g/100g]	K [Bq/kg]	Cs [Bq/kg]
		Nutrient contents and radioactivity concentrations								
Yamagata	1	65	4.0	1.2	9.6	10	83.7	1.5	1190.95	9.97
	2	66	4.2	1.2	9.5	10	83.5	1.6	-	-
	3	66	4.3	1.2	9.5	11	83.5	1.5	-	-
	Mean ± SD (*n* = 3)	65.67 ± 0.47	4.167 ± 0.125	1.200 ± 0	9.533 ± 0.047	10.33 ± 0.47	83.567 ± 0.094	1.533 ± 0.047	-	-
Sendai-1	1	72	3.7	1.2	11.7	9	82.0	1.4	719.66	ND
	2	72	3.9	1.1	11.5	9	82.1	1.4	-	-
	3	72	3.8	1.1	11.6	9	82.1	1.4	-	-
	Mean ± SD (*n* = 3)	72.00 ± 0	3.800 ± 0.082	1.133 ± 0.047	11.600 ± 0.082	9.00 ± 0	82.067 ± 0.047	1.400 ± 0	-	-
Sendai-2	1	60	3.9	0.8	9.9	4	84.2	1.1	819.12	8.41
	2	62	3.8	0.8	9.8	4	84.5	1.1	-	-
	3	63	3.8	0.9	9.4	4	84.9	1.1	-	-
	Mean ± SD (*n* = 3)	61.67 ± 1.25	3.833 ± 0.047	0.833 ± 0.047	9.700 ± 0.216	4.00 ± 0	84.533 ± 0.287	1.100 ± 0	-	-
Ryozen	1	75	4.2	1.3	11.7	4	81.6	1.2	653.99	66.16
	2	76	4.2	1.2	12.0	5	81.4	1.2	-	-
	3	77	4.3	1.3	11.9	4	81.3	1.2	-	-
	Mean ± SD (*n* = 3)	76.00 ± 0.82	4.233 ± 0.047	1.267 ± 0.047	11.867 ± 0.125	4.33 ± 0.47	81.433 ± 0.125	1.200 ± 0	-	-
Tomioka-1	1	61	3.2	1.0	9.7	7	84.6	1.5	879.29	564.49
	2	60	3.2	1.0	9.5	7	84.8	1.5	-	-
	3	60	3.3	0.9	9.6	7	84.7	1.5	-	-
	Mean ± SD (*n* = 3)	60.33 ± 0.47	3.233 ± 0.047	0.967 ± 0.047	9.600 ± 0.082	7.00 ± 0	84.700 ± 0.082	1.500 ± 0	-	-
Iwaki-1	1	61	3.8	1.3	8.6	8	84.9	1.4	852.81	17.38
	2	60	3.9	1.2	8.5	8	85.0	1.4	-	-
	3	60	3.9	1.1	8.6	8	85.0	1.4	-	-
	Mean ± SD (*n* = 3)	60.33 ± 0.47	3.867 ± 0.047	1.200 ± 0.082	8.567 ± 0.047	8.00 ± 0	84.967 ± 0.047	1.400 ± 0	-	-
Minamisoma	1	59	3.2	1.0	9.2	3	85.7	1.0	691.00	28.90
	2	58	3.2	0.9	9.2	3	85.7	1.0	-	-
	3	58	3.2	0.9	9.2	3	85.6	1.0	-	-
	Mean ± SD (*n* = 3)	58.33 ± 0.47	3.200 ± 0	0.933 ± 0.047	9.200 ± 0	3.00 ± 0	85.667 ± 0.047	1.000 ± 0	-	-
Namie-1	1	59	3.6	1.0	9.0	4	85.2	1.1	860.58	328.06
	2	59	3.6	1.0	8.8	4	85.5	1.1	-	-
	3	59	3.6	1.0	9.0	4	85.3	1.2	-	-
	Mean ± SD (*n* = 3)	59.00 ± 0	3.600 ± 0	1.000 ± 0	8.933 ± 0.094	4.00 ± 0	85.333 ± 0.125	1.133 ± 0.047	-	-

Table S2. *Continued.*

City/Town	Replication, mean ± SD (*n*)	Nutrient contents and radioactivity concentrations								
		EN [kcal/100g]	PR [g/100g]	LI [g/100g]	CA [g/100g]	Na [mg/100g]	WA [g/100g]	AS [g/100g]	K [Bq/kg]	Cs [Bq/kg]
Soma	1	55	3.3	0.9	8.6	3	86.1	1.1	550.98	39.19
	2	55	3.3	0.9	8.3	3	86.4	1.1	-	-
	3	56	3.3	0.9	8.5	3	86.2	1.1	-	-
	Mean ± SD (*n* = 3)	55.33 ± 0.47	3.300 ± 0	0.900 ± 0	8.467 ± 0.125	3.00 ± 0	86.233 ± 0.125	1.100 ± 0	-	-
Iitate-1	1	60	3.0	0.8	10.1	1	85.1	0.9	719.69	257.78
	2	61	2.9	0.9	10.4	1	84.9	0.9	-	-
	3	61	2.9	0.9	10.2	2	85.2	0.9	-	-
	Mean ± SD (*n* = 3)	60.67 ± 0.47	2.933 ± 0.047	0.867 ± 0.047	10.233 ± 0.125	1.33 ± 0.47	85.067 ± 0.125	0.900 ± 0	-	-
Iitate-2	1	61	3.5	0.8	9.7	2	84.9	1.0	511.17	300.37
	2	61	3.6	0.8	9.8	1	84.8	1.0	-	-
	3	61	3.6	0.9	9.8	1	84.8	1.0	-	-
	Mean ± SD (*n* = 3)	61.00 ± 0	3.567 ± 0.047	0.833 ± 0.047	9.767 ± 0.047	1.33 ± 0.47	84.833 ± 0.047	1.000 ± 0	-	-
Namie-2	1	57	3.6	1.0	9.0	3	85.2	1.3	617.14	211.32
	2	59	3.6	0.9	9.0	3	85.3	1.2	-	-
	3	59	3.6	1.0	8.4	3	85.7	1.2	-	-
	Mean ± SD (*n* = 3)	58.33 ± 0.94	3.600 ± 0	0.967 ± 0.047	8.800 ± 0.283	3.00 ± 0	85.400 ± 0.216	1.233 ± 0.047	-	-
Tomioka-2	1	62	3.5	1.0	9.7	3	84.4	1.4	580.89	198.08
	2	60	3.4	0.8	9.8	3	84.5	1.5	-	-
	3	61	3.4	0.9	9.8	3	84.4	1.5	-	-
	Mean ± SD (*n* = 3)	61.00 ± 0.82	3.433 ± 0.047	0.900 ± 0.082	9.767 ± 0.047	3.00 ± 0	84.433 ± 0.047	1.467 ± 0.047	-	-
Iwaki-2	1	57	3.5	0.9	8.8	7	85.8	1.0	757.34	15.56
	2	57	3.6	0.9	8.6	7	85.9	1.0	-	-
	3	57	3.5	0.9	8.7	7	85.9	1.0	-	-
	Mean ± SD (*n* = 3)	57.00 ± 0	3.533 ± 0.047	0.900 ± 0	8.700 ± 0.082	7.00 ± 0	85.867 ± 0.047	1.000 ± 0	-	-

Note: EN: energy, PR: protein, LI: lipid, CA: carbohydrate, Na: sodium, WA: water, AS: ash, K: ^{40}K (potassium-40), Cs: ^{137}Cs (cesium-137). -: Not applicable, ND: Not determined (below detection limit). For mean and SD values, two digits after significant digits were saved just for reference.

Table S3. Nutrient contents and ^{40}K and ^{137}Cs radioactivity concentrations in *Oxalis* leaves in the Niigata group.

City/Town	Replication, mean ± SD (*n*)	Nutrient contents and radioactivity concentrations								
		EN [kcal/100g]	PR [g/100g]	LI [g/100g]	CA [g/100g]	Na [mg/100g]	WA [g/100g]	AS [g/100g]	K [Bq/kg]	Cs [Bq/kg]
Joetsu	1	69	3.7	1.0	11.4	11	82.5	1.4	908.18	ND
	2	70	3.8	1.0	11.4	11	82.4	1.4	-	-
	Mean ± SD (*n* = 2)	69.50 ± 0.50	3.750 ± 0.050	1.000 ± 0	11.400 ± 0	11.00 ± 0	82.450 ± 0.050	1.400 ± 0	-	-
Kashiwazaki	1	59	3.7	1.0	8.8	12	85.2	1.3	899.00	ND
	2	60	3.8	1.0	8.9	12	85.0	1.3	-	-
	Mean ± SD (*n* = 2)	59.50 ± 0.50	3.750 ± 0.050	1.000 ± 0	8.850 ± 0.050	12.00 ± 0	85.100 ± 0.100	1.300 ± 0	-	-
Murakami	1	67	4.4	1.3	9.5	23	83.3	1.5	965.80	ND
	2	67	4.0	1.3	9.9	23	83.3	1.5	-	-
	Mean ± SD (*n* = 2)	67.00 ± 0	4.200 ± 0.200	1.300 ± 0	9.700 ± 0.200	23.00 ± 0	83.300 ± 0	1.500 ± 0	-	-
Niigata	1	64	3.9	1.2	9.4	18	84.1	1.4	961.16	ND
	2	62	3.8	1.2	9.1	17	84.5	1.4	-	-
	Mean ± SD (*n* = 2)	63.00 ± 1.00	3.850 ± 0.050	1.200 ± 0	9.250 ± 0.150	17.50 ± 0.50	84.300 ± 0.200	1.400 ± 0	-	-
Yahiko	1	75	4.1	1.5	11.2	32	81.6	1.6	927.00	ND
	2	75	4.3	1.5	11.0	30	81.6	1.6	-	-
	Mean ± SD (*n* = 2)	75.00 ± 0	4.200 ± 0.100	1.500 ± 0	11.100 ± 0.100	31.00 ± 1.00	81.600 ± 0	1.600 ± 0	-	-

Note: EN: energy, PR: protein, LI: lipid, CA: carbohydrate, Na: sodium, WA: water, AS: ash, K: ^{40}K (potassium-40), Cs: ^{137}Cs (cesium-137). -: Not applicable, ND: Not determined (below detection limit). For mean and SD values, two digits after significant digits were saved just for reference.

Table S4. Nutrient contents and ^{40}K and ^{137}Cs radioactivity concentrations in *Oxalis* leaves in the Kyushu group.

City/Town	Replication, mean ± SD (*n*)	Nutrient contents and radioactivity concentrations								
		EN [kcal/100g]	PR [g/100g]	LI [g/100g]	CA [g/100g]	Na [mg/100g]	WA [g/100g]	AS [g/100g]	K [Bq/kg]	Cs [Bq/kg]
Satsumasendai	1	72	3.6	1.3	11.5	14	82.1	1.5	777.02	ND
	2	72	3.6	1.3	11.4	14	82.2	1.5	-	-
	3	72	3.6	1.3	11.4	14	82.2	1.5	-	-
	Mean ± SD (*n* = 3)	72.00 ± 0	3.600 ± 0	1.300 ± 0	11.433 ± 0.047	14.00 ± 0	82.167 ± 0.047	1.500 ± 0	-	-
Izumi	1	62	3.7	1.2	9.1	8	83.4	2.6	969.25	ND
	2	61	3.8	1.1	8.9	8	83.6	2.6	-	-
	3	61	3.8	1.1	9.0	8	83.5	2.6	-	-
	Mean ± SD (*n* = 3)	61.33 ± 0.47	3.767 ± 0.047	1.133 ± 0.047	9.000 ± 0.082	8.00 ± 0	83.500 ± 0.082	2.600 ± 0	-	-
Kumamoto	1	54	3.2	1.0	8.0	8	85.4	2.4	737.21	ND
	2	52	3.3	1.0	7.5	8	85.8	2.4	-	-
	3	53	3.2	1.0	7.8	8	85.5	2.5	-	-
	Mean ± SD (*n* = 3)	53.00 ± 0.82	3.233 ± 0.047	1.000 ± 0	7.767 ± 0.205	8.00 ± 0	85.567 ± 0.170	2.433 ± 0.047	-	-
Yatsushiro	1	65	3.5	1.1	10.3	8	83.6	1.5	840.27	ND
	2	66	3.5	1.1	10.4	8	83.4	1.6	-	-
	3	65	3.5	1.0	10.6	9	83.3	1.6	-	-
	Mean ± SD (*n* = 3)	65.33 ± 0.47	3.500 ± 0	1.067 ± 0.047	10.433 ± 0.125	8.33 ± 0.47	83.433 ± 0.125	1.567 ± 0.047	-	-
Nishihara-AM	1	60	3.6	1.1	8.8	13	85.0	1.5	931.66	ND
	2	60	3.7	1.2	8.6	13	85.0	1.5	-	-
	3	60	3.6	1.1	9.0	13	84.8	1.5	-	-
Nishihara-PM	1	67	3.6	1.3	10.3	16	83.2	1.6	-	-
	2	67	3.7	1.3	10.2	16	83.2	1.6	-	-
	3	67	3.6	1.2	10.5	16	83.1	1.6	-	-
	Mean ± SD (*n* = 6)	63.50 ± 3.50	3.633 ± 0.047	1.200 ± 0.082	9.567 ± 0.780	14.50 ± 1.50	84.050 ± 0.886	1.540 ± 0.050	-	-

Note: EN: energy, PR: protein, LI: lipid, CA: carbohydrate, Na: sodium, WA: water, AS: ash, K: ^{40}K (potassium-40), Cs: ^{137}Cs (cesium-137). -: Not applicable, ND: Not determined (below detection limit). For mean and SD values, two digits after significant digits were saved just for reference.

Table S5. Pearson correlation coefficients and their associated *p*-values among nutrient contents, ^{40}K and ^{137}Cs radioactivity concentrations in *Oxalis* leaves, temperature, and ground radiation dose in 3 groups combined ($n = 24$).

	EN	PR	LI	CA	Na	WA	AS	K	Cs	Temperature
PR	$r = 0.65$<br>$p = 5.4\times10^{-4}$***									
LI	$r = 0.71$<br>$p = 9.9\times10^{-5}$**	$r = 0.71$<br>$p = 1.1\times10^{-4}$***								
CA	$r = 0.92$<br>$p = 2.8\times10^{-10}$*	$r = 0.35$<br>$p = 0.094$	$r = 0.41$<br>$p = 0.048$*							
Na	$r = 0.52$<br>$p = 0.0090$**	$r = 0.59$<br>$p = 0.0026$**	$r = 0.81$<br>$p = 1.3\times10^{-6}$**	$r = 0.25$<br>$p = 0.23$						
WA	$r = -0.97$<br>$p = 1.0\times10^{-14}$**	$r = -0.64$<br>$p = 6.9\times10^{-4}$***	$r = -0.72$<br>$p = 6.6\times10^{-5}$**	$r = -0.87$<br>$p = 2.5\times10^{-8}$**	$r = -0.53$<br>$p = 0.0072$**					
AS	$r = 0.0050$<br>$p = 0.98$	$r = 0.13$<br>$p = 0.54$	$r = 0.37$<br>$p = 0.077$	$r = -0.16$<br>$p = 0.45$	$r = 0.31$<br>$p = 0.13$	$r = -0.24$<br>$p = 0.26$				
K	$r = 0.24$<br>$p = 0.26$	$r = 0.49$<br>$p = 0.015$*	$r = 0.53$<br>$p = 0.0083$**	$r = 0.0036$<br>$p = 0.99$	$r = 0.59$<br>$p = 0.0023$**	$r = -0.30$<br>$p = 0.16$	$r = 0.38$<br>$p = 0.063$			
Cs	$r = -0.26$<br>$p = 0.21$	$r = -0.44$<br>$p = 0.031$*	$r = -0.42$<br>$p = 0.039$*	$r = -0.086$<br>$p = 0.69$	$r = -0.43$<br>$p = 0.038$*	$r = 0.30$<br>$p = 0.15$	$r = -0.27$<br>$p = 0.21$	$r = -0.28$<br>$p = 0.18$		
Temperature	$r = -0.021$<br>$p = 0.92$	$r = 0.17$<br>$p = 0.44$	$r = 0.071$<br>$p = 0.74$	$r = -0.093$<br>$p = 0.67$	$r = 0.46$<br>$p = 0.022$*	$r = 0.091$<br>$p = 0.67$	$r = -0.23$<br>$p = 0.27$	$r = 0.16$<br>$p = 0.46$	$r = -0.16$<br>$p = 0.44$	
Ground dose	$r = -0.29$<br>$p = 0.16$	$r = -0.35$<br>$p = 0.099$	$r = -0.41$<br>$p = 0.049$*	$r = -0.16$<br>$p = 0.47$	$r = -0.48$<br>$p = 0.018$*	$r = 0.36$<br>$p = 0.080$	$r = -0.38$<br>$p = 0.070$	$r = -0.42$<br>$p = 0.040$*	$r = 0.63$<br>$p = 9.6\times10^{-4}$***	$r = 0.075$<br>$p = 0.73$

Note: EN: energy, PR: protein, LI: lipid, CA: carbohydrate, Na: sodium, WA: water, AS: ash, K: ^{40}K (potassium-40), CS: ^{137}Cs (cesium-137). ND (not determined) measurements were considered zero to obtain the values in this table. LI vs Cs: $r = -0.48$, $p = 0.022$*, Na vs Cs: $r = -0.53$, $p = 0.0088$**, and K vs Cs: $r = -0.49$, $p = 0.017$* without Tomioka-1. *: $p < 0.05$, **: $p < 0.01$, ***: $p < 0.001$.

Table S6. Pearson correlation coefficients and their associated *p*-values among nutrient contents, ^{40}K and ^{137}Cs radioactivity concentrations in *Oxalis* leaves, temperature, and ground radiation dose in the Tohoku group (n = 14).

	EN	PR	LI	CA	Na	WA	AS	K	Cs	Temperature
PR	r = 0.66<br>p = 0.011*									
LI	r = 0.69<br>p = 0.0059**	r = 0.73<br>p = 0.0031**								
CA	r = 0.91<br>p = 4.7×10^{-6}***	r = 0.33<br>p = 0.24	r = 0.39<br>p = 0.17							
Na	r = 0.35<br>p = 0.23	r = 0.54<br>p = 0.046*	r = 0.66<br>p = 0.0098**	r = 0.084<br>p = 0.78						
WA	r = -0.99<br>p = 4.1×10^{-12}***	r = -0.66<br>p = 0.0097**	r = -0.68<br>p = 0.0072**	r = -0.91<br>p = 8.2×10^{-6}***	r = -0.38<br>p = 0.18					
AS	r = 0.33<br>p = 0.24	r = 0.43<br>p = 0.12	r = 0.57<br>p = 0.033*	r = 0.13<br>p = 0.66	r = 0.68<br>p = 0.0071**	r = -0.43<br>p = 0.13				
K	r = 0.13<br>p = 0.66	r = 0.38<br>p = 0.18	r = 0.45<br>p = 0.10	r = -0.092<br>p = 0.75	r = 0.74<br>p = 0.0026**	r = -0.15<br>p = 0.60	r = 0.47<br>p = 0.093			
Cs	r = -0.23<br>p = 0.44	r = -0.47<br>p = 0.093	r = -0.31<br>p = 0.28	r = -0.056<br>p = 0.85	r = -0.30<br>p = 0.30	r = 0.19<br>p = 0.52	r = 0.083<br>p = 0.78	r = -0.070<br>p = 0.81		
Temperature	r = -0.69<br>p = 0.0061**	r = -0.48<br>p = 0.081	r = -0.69<br>p = 0.0062**	r = -0.56<br>p = 0.038*	r = -0.63<br>p = 0.015*	r = 0.71<br>p = 0.0044**	r = -0.57<br>p = 0.034*	r = -0.37<br>p = 0.19	r = 0.083<br>p = 0.78	
Ground dose	r = -0.28<br>p = 0.32	r = -0.33<br>p = 0.24	r = -0.30<br>p = 0.29	r = -0.17<br>p = 0.56	r = -0.57<br>p = 0.032*	r = 0.29<br>p = 0.31	r = -0.28<br>p = 0.32	r = -0.28<br>p = 0.33	r = 0.52<br>p = 0.058	r = 0.54<br>p = 0.049*

Note: EN: energy, PR: protein, LI: lipid, CA: carbohydrate, Na: sodium, WA: water, AS: ash, K: ^{40}K (potassium-40), Cs: ^{137}Cs (cesium-137). ND (not determined) measurements were considered zero to obtain the values in this table. LI vs Cs: r = -0.39, p = 0.19, Na vs Cs: r = -0.65, p = 0.016*, K vs Cs: r = -0.33, p = 0.27, Cs vs Ground dose: r = 0.86, p = 1.5×10^{-4}***, and Cs vs Temperature: r = 0.41, p = 0.16 without Tomioka-1. *: $p < 0.05$, **: $p < 0.01$, ***: $p < 0.001$.

Table S7. Pearson correlation coefficients and their associated *p*-values among nutrient contents, ^{40}K radioactivity concentration in *Oxalis* leaves, temperature, and ground radiation dose in the Niigata group (n = 5).

	EN	PR	LI	CA	Na	WA	AS	K	Temperature
PR	r = 0.61<br>p = 0.27								
LI	r = 0.67<br>p = 0.21	r = 0.91<br>p = 0.029*							
CA	r = 0.89<br>p = 0.044*	r = 0.21<br>p = 0.73	r = 0.26<br>p = 0.67						
Na	r = 0.68<br>p = 0.21	r = 0.93<br>p = 0.022*	r = 0.99<br>$p = 4.8\times10^{-4}$***	r = 0.27<br>p = 0.66					
WA	r = -0.99<br>$p = 6.7\times10^{-4}$***	r = -0.55<br>p = 0.34	r = -0.59<br>p = 0.30	r = -0.93<br>p = 0.022*	r = -0.60<br>p = 0.29				
AS	r = 0.88<br>p = 0.050*	r = 0.90<br>p = 0.038*	r = 0.93<br>p = 0.022*	r = 0.57<br>p = 0.32	r = 0.93<br>p = 0.022*	r = -0.83<br>p = 0.082			
K	r = -0.014<br>p = 0.98	r = 0.50<br>p = 0.39	r = 0.45<br>p = 0.44	r = -0.28<br>p = 0.65	r = 0.39<br>p = 0.52	r = 0.044<br>p = 0.94	r = 0.35<br>p = 0.57		
Temperature	r = -0.65<br>p = 0.23	r = 0.12<br>p = 0.85	r = -0.16<br>p = 0.80	r = -0.79<br>p = 0.11	r = -0.14<br>p = 0.82	r = 0.67<br>p = 0.22	r = -0.32<br>p = 0.59	r = 0.38<br>p = 0.53	
Ground dose	r = 0.10<br>p = 0.87	r = 0.53<br>p = 0.35	r = 0.34<br>p = 0.58	r = -0.074<br>p = 0.91	r = 0.29<br>p = 0.63	r = -0.11<br>p = 0.86	r = 0.37<br>p = 0.54	r = 0.90<br>p = 0.038*	r = 0.43<br>p = 0.47

Note: EN: energy, PR: protein, LI: lipid, CA: carbohydrate, Na: sodium, WA: water, AS: ash, K: ^{40}K (potassium-40). *: $p < 0.05$, ***: $p < 0.001$.

Table S8. Pearson correlation coefficients and their associated *p*-values among nutrient contents, ^{40}K radioactivity concentration in *Oxalis* leaves, temperature, and ground radiation dose in the Kyushu group (n = 5).

	EN	PR	LI	CA	Na	WA	AS	K	Temperature
PR	r = 0.58<br>p = 0.31								
LI	r = 0.85<br>p = 0.071	r = 0.64<br>p = 0.24							
CA	r = 0.98<br>p = 0.0024**	r = 0.45<br>p = 0.45	r = 0.76<br>p = 0.14						
Na	r = 0.62<br>p = 0.26	r = 0.32<br>p = 0.60	r = 0.85<br>p = 0.071	r = 0.56<br>p = 0.32					
WA	r = -0.96<br>p = 0.0089**	r = -0.68<br>p = 0.21	r = -0.81<br>p = 0.096	r = -0.93<br>p = 0.024*	r = -0.46<br>p = 0.44				
AS	r = -0.74<br>p = 0.15	r = -0.12<br>p = 0.85	r = -0.56<br>p = 0.33	r = -0.80<br>p = 0.11	r = -0.71<br>p = 0.18	r = 0.54<br>p = 0.35			
K	r = 0.11<br>p = 0.86	r = 0.83<br>p = 0.084	r = 0.19<br>p = 0.76	r = -0.017<br>p = 0.98	r = 0.057<br>p = 0.93	r = -0.19<br>p = 0.76	r = 0.12<br>p = 0.84		
Temperature	r = 0.53<br>p = 0.36	r = 0.52<br>p = 0.37	r = 0.73<br>p = 0.16	r = 0.44<br>p = 0.45	r = 0.91<br>p = 0.034*	r = -0.37<br>p = 0.54	r = -0.65<br>p = 0.23	r = 0.43<br>p = 0.47	
Ground dose	r = 0.56<br>p = 0.33	r = 0.83<br>p = 0.082	r = 0.36<br>p = 0.55	r = 0.50<br>p = 0.40	r = -0.11<br>p = 0.86	r = -0.73<br>p = 0.16	r = -0.0049<br>p = 0.99	r = 0.62<br>p = 0.27	r = 0.038<br>p = 0.95

Note: EN: energy, PR: protein, LI: lipid, CA: carbohydrate, Na: sodium, WA: water, AS: ash, K: ^{40}K (potassium-40). *: $p < 0.05$, **: $p < 0.01$.

Table S9. Coefficient of variation (CV) among nutrient contents and ^{40}K radioactivity concentration in *Oxalis* leaves.

Group		EN	PR	LI	CA	Na	WA	AS	K
3 groups	SD	5.93	0.334	0.171	1.073	6.97	1.370	0.393	155.89
	Mean	63.16	3.658	1.067	9.718	8.99	84.149	1.408	805.05
	CV	0.0939	0.0912	0.161	0.1104	0.776	0.01628	0.279	0.19365
Tohoku	SD	5.51	0.354	0.141	1.000	2.76	1.324	0.205	168.09
	Mean	61.90	3.593	0.993	9.624	4.88	84.579	1.212	743.19
	CV	0.0889	0.0984	0.142	0.1039	0.565	0.01566	0.168	0.22618
Niigata	SD	5.33	0.207	0.190	1.013	7.42	1.252	0.102	27.11
	Mean	66.80	3.950	1.200	10.060	18.90	83.350	1.440	932.23
	CV	0.0799	0.0525	0.158	0.1007	0.393	0.01502	0.0708	0.02908
Kyushu	SD	6.16	0.179	0.104	1.246	3.01	1.101	0.483	88.35
	Mean	63.03	3.545	1.140	9.640	10.57	83.743	1.928	851.08
	CV	0.0977	0.0503	0.0913	0.1293	0.285	0.01315	0.251	0.1038

Note: EN: energy, PR: protein, LI: lipid, CA: carbohydrate, Na: sodium, WA: water, AS: ash, K: ^{40}K (potassium-40).

Table S10. Shapiro-Wilk test for normality (*p*-values) using raw data, ratio transformed (rt) data, and centered log-ratio transformed (clr) data.

	Group	EN	PR	LI	CA	Na	WA	AS	K	Cs	Temperature	Ground dose
Raw	3 groups	0.12	0.49	0.13	0.15	0.0024	0.14	$4.6{\times}10^{-4}$	0.74	-	0.24	-
	Tohoku	0.0067	0.94	0.039	0.038	0.13	0.033	0.21	0.20	0.0087	0.12	0.0032
	Niigata	0.98	0.048	0.47	0.38	0.56	0.94	0.81	0.25	-	0.45	-
	Kyushu	0.91	0.66	0.98	1.0	0.020	0.79	0.041	0.67	-	0.57	-
Rt	3 groups	-	0.79	0.31	0.98	-	0.33	$3.3{\times}10^{-5}$	-	-	-	-
	Tohoku	-	0.95	0.53	0.99	-	0.20	0.30	-	-	-	-
	Niigata	-	0.38	0.82	0.046	-	0.98	0.42	-	-	-	-
	Kyushu	-	0.71	0.46	0.53	-	0.37	0.062	-	-	-	-
Clr	3 groups	-	0.67	0.47	0.86	-	0.42	0.0012	-	-	-	-
	Tohoku	-	0.90	0.6	1.0	-	0.14	0.42	-	-	-	-
	Niigata	-	0.37	0.78	0.061	-	0.96	0.47	-	-	-	-
	Kyushu	-	0.69	0.48	0.51	-	0.35	0.080	-	-	-	-

Note: EN: energy, PR: protein, LI: lipid, CA: carbohydrate, Na: sodium, WA: water, AS: ash, K: ^{40}K (potassium-40), Cs: ^{137}Cs (cesium-137).
-: Not applicable. Cs: p = 0.0079 without Tomioka in the Tohoku Group.

Table S11. Pearson correlation coefficients and their associated *p*-values between raw data and ratio transformed (rt) data and between raw data and centered log-ratio transformed (clr) data.

		PR	LI	CA	WA	AS
Raw vs Rt	3 groups	r = 0.49<br>p = 0.015*	r = 0.91<br>$p = 6.1\times10^{-10}$***	r = 0.69<br>$p = 1.9\times10^{-4}$***	r = 0.83<br>$p = 4.8\times10^{-7}$***	r = 0.97<br>$p = 2.3\times10^{-15}$***
	Tohoku	r = 0.64<br>p = 0.013*	r = 0.90<br>$p = 8.8\times10^{-6}$***	r = 0.69<br>p = 0.0066**	r = 0.86<br>$p = 9.3\times10^{-5}$***	r = 0.93<br>$p = 1.4\times10^{-6}$***
	Niigata	r = -0.097<br>p = 0.88	r = 0.98<br>p = 0.0047**	r = 0.80<br>p = 0.11	r = 0.89<br>p = 0.045*	r = 0.91<br>p = 0.033*
	Kyushu	r = 0.66<br>p = 0.22	r = 0.92<br>p = 0.026*	r = 0.96<br>p = 0.0085**	r = 0.68<br>p = 0.21	r = 0.99<br>p = 0.0021**
Raw vs Clr	3 groups	r = 0.49<br>p = 0.015*	r = 0.90<br>$p = 1.3\times10^{-9}$***	r = 0.69<br>$p = 1.9\times10^{-4}$***	r = 0.84<br>$p = 2.8\times10^{-7}$***	r = 0.97<br>$p = 1.1\times10^{-15}$***
	Tohoku	r = 0.65<br>p = 0.013*	r = 0.89<br>$p = 1.6\times10^{-5}$***	r = 0.68<br>p = 0.0069**	r = 0.87<br>$p = 5.8\times10^{-5}$***	r = 0.93<br>$p = 1.1\times10^{-6}$***
	Niigata	r = -0.095<br>p = 0.88	r = 0.97<br>p = 0.0075**	r = 0.80<br>p = 0.10	r = 0.89<br>p = 0.046*	r = 0.93<br>p = 0.022*
	Kyushu	r = 0.67<br>p = 0.22	r = 0.92<br>p = 0.029*	r = 0.96<br>p = 0.0090**	r = 0.67<br>p = 0.22	r = 0.99<br>p = 0.0022**

Note: PR: protein, LI: lipid, CA: carbohydrate, WA: water, AS: ash. *: $p < 0.05$, **: $p < 0.01$, ***: $p < 0.001$.
